# The uropygial gland of the European hoopoe as a symbiotic organ

**DOI:** 10.1186/s42523-026-00543-y

**Published:** 2026-04-30

**Authors:** Manuel Martín-Vivaldi, Ángela Martínez-García, Juan M. Peralta-Sánchez, Michael Schaub, Raphaël Arlettaz, Antonio M. Martín-Platero, Ester Martínez-Renau, María Dolores Barón, Magdalena Ruiz-Rodríguez, Estefanía López-Hernández, Manuel Martínez-Bueno, Eva Valdivia, Juan J. Soler

**Affiliations:** 1 Departamento de Zoología, Universidad de Granada, Granada, 18071 Spain; 2 IES Padre Manjón, Granada, 18003 Spain; 3 Departamento de Zoología, Universidad de Sevilla, Sevilla, 41012 Spain; 4 Swiss Ornithological Institute, Sempach, 6204 Switzerland; 5 Division of Conservation Biology, Institute of Ecology and Evolution, University of Bern, Bern, 3012 Switzerland; 6 Departamento de Microbiología, Universidad de Granada, Granada, 18071 Spain; 7 Department of Ecology and Genetics, Uppsala University, Uppsala, Sweden; 8 Departamento de Ecología Funcional y Evolutiva, Estación Experimental de Zonas Áridas (CSIC), Almería, 04120 Spain; 9 Coevolución: Cucos, Hospedadores y Bacterias Simbiontes, Unidad Asociada (CSIC), Universidad de Granada, Granada, 18071 Spain

**Keywords:** ARISA, Bacterial community, Coevolution, Geographic variation, Illumina, Mutualistic symbiosis, Uropygial secretion, Symbiotic organs

## Abstract

**Background:**

Animals rely on symbiotic bacteria living within/on their tissues for multiple functions, while simultaneously needing to protect themselves via immune functions or other defenses from potentially pathogenic microorganisms that could invade those tissues. As a result, interactions with complex assemblages of bacteria have driven the evolution of host strategies to control established symbioses. One such strategy involves the development of organs specialized in maintaining associations with beneficial members of the microbial community — so-called symbiotic organs. These organs are characterized by compartmentalizing spaces where favorable conditions for the beneficial bacteria are promoted, while preventing colonization of other tissues. Although several model systems of symbiotic organs have been studied in animals, none have been recognized in non-aquatic vertebrates except for the intestinal crypts of mammals. Here, we propose that bird´s uropygial glands may be specialized symbiotic organs.

**Results:**

We tested this hypothesis using the uropygial gland of the hoopoe (*Upupa epops*) as a model, which hosts a complex bacterial community that includes antimicrobial-producing symbionts. First, we examined whether the uropygial gland supports a specific symbiotic community, by comparing the microbiome composition of the uropygial secretions and eggshell surfaces in two European hoopoe populations. Additionally, using histological staining and fluorescence in situ hybridization, we looked for structural specializations for compartmentalization and bacterial targeting in the glands of nesting hoopoes in comparison to non-breeding individuals lacking the symbiosis. Results show that, in comparison with bacterial communities of the eggshells, those of the uropygial gland were more conserved between both populations. Moreover, uropygial glands of nesting hoopoes were strictly compartmentalized by a special tissue, properties that are absent in the non-breeding individuals lacking the symbiosis. Finally, bacteria were organized within the organ, suggesting the existence of special physical niches to promote specialized mutualistic symbionts.

**Conclusions:**

All evidence supports that the hoopoe uropygial gland is a specialized *symbiotic organ* for bacterial cultivation, paving the way for new insights into vertebrates’ exocrine glands’ role in microbial symbiosis.

**Clinical trial number:**

Not applicable.

**Supplementary Information:**

The online version contains supplementary material available at 10.1186/s42523-026-00543-y.

## Background

Bacterial symbiosis is currently recognized as one of the main interactions driving evolution of plants and animals, including vertebrates [1]. External and internal epithelial tissues of healthy animals harbor a diversity of microbial assemblages that are consistent across species [2] and populations [3, 4], while also showing individual specificity [5, 6]. The microbiome expands host functionality [7] with stability [8], but also presents adaptability [9, 10], thus facilitating plastic responses to environmental conditions.

The importance of symbiotic bacteria for their animal hosts [11] ranges from providing nutrients [12, 13], to enhancing pathogen resistance by promoting immune response [14], or producing defensive substances [15–21]. Symbionts also play central roles in thermal tolerance [22], sensorial capacity [23], social behavior [24, 25], or mental health [26, 27]. Host traits that favor the acquisition of adequate or optimal symbionts for these functions would increase host fitness [28, 29] and, thus, evolve as adaptations in host populations [30–32]. In this sense, host traits assuring vertical transmission of beneficial microbes have traditionally been recognized as adaptations that ensure the acquisition of the optimal symbionts [33–37]. Highly specific mutualisms can also evolve in the absence of vertical transmission. For example *Drosophila* fruit flies acquire symbionts from the environment to their simple gut microbiomes [38], and the *Euprymna scolopes* squid, recruits *Vibrio fisheri* fluorescent inhabitants from the environment to its luminescent organ [39, 40]. Indeed, it is progressively accepted that, from the hundreds of bacterial strains that come into contact with animals, with apparent stochastic transit, hosts are able to select, maintain and promote particular species or communities ([41], Box 1). Such selection occurs in sites specialized for colonization and growth of those symbionts [42–44]. Favoring *immigration*, *compartmentalization*, *monitoring* and *targeting* (Box 1), hosts can exert *partner choice* (Box 1) and control the symbiont community established [32]. Among host characteristics favoring acquisition of the “chosen” symbionts, the compartmentalization of body regions allows cultivating bacteria in particular locations where the hosts control the chemical and physical environment (thus allowing specific *monitoring* and *targeting*, Box 1). Such compartmentalization strategies would involve the physical delimitation of spaces to promote the establishment and growth of specific symbionts. This can be achieved by facilitating or penalizing bacteria depending on their associated benefits [45]. Examples of hosts conferring these strategies include insect bacteriocytes housing endosymbionts [46–48], or the root nodules of legumes. In the latter, the plant monitors the level of N_2_ fixation by the single bacterial strain responsible for each nodule and decides nutrient rewards or abortion accordingly [49]. A variety of animal cavities also function as physical compartments where mutualistic microbiomes of various levels of complexity are housed. This is the case of luminescent squid crypts [40], the pouches/crypts in the wall of the animals’ gut [50–52] or the cuticle of some ant species [53, 54]. These *physical niches* for symbionts might also be more fluid [45] compartments in time and space, as in the foregut of fruit flies [55]. We are at the beginning of understanding how animals establish mutualistic associations with bacteria, and discovering new special host compartments that provide appropriate niches for bacteria [56].

**Box 1:** Glossary of terms used in the context of control of host-symbionts associations

Term	Meaning	Source
Partner choice	Active behavioral response in one species that favors cooperation in the other. This is achieved either: (i) interacting more with cooperators, (ii) interacting randomly but rejecting non-cooperators, (iii) interacting randomly but supplying less aid to non-cooperators.	Foster & Wenseleers 2006 [41]
Favoring immigration	Control exerted by host to influence which microbial strains and species it encounters (in the environment) or are acquired by progeny (vertical transmission).	Foster et al. 2017 [32]
Compartmentalization	Physical and chemical barriers (such as epithelia and associated mucus layers) in/on hosts that limit accessibility of microbes to particular tissues or cavities, getting germ-free body locations and separating microbes in space.	Foster et al. 2017 [32]; Chomicki et al. 2020 [45]
Monitoring	Host detects the location and identity/effects of microbes within its body, either with adaptive immunity/toll-like receptors of innate immunity, or by *measuring* the net income of beneficial resources obtained from symbionts.	Foster et al. 2017 [32]
Targeting	Host promotion of the right symbionts by providing specific resources they need (positive selection) or secreting specific antimicrobials against undesired invaders (negative selection).	Foster et al. 2017 [32]
Symbiotic organ	Host structures that house populations of beneficial microbes providing a core set of mutualism services to hosts. Microbial communities are filtered by symbiotic organs, mediating which genotypes interact with hosts, gain host resources, and are subsequently passed on to new hosts (see Box 2).	Fronk and Sachs 2022 [57]
Physical niche	A host-constructed niche provides specific sites for adhesion, protected space that limits loss of bacteria, and a specifically tailored nutritional and immune environment that selects the proper strains from the available microbiota.	Ludington 2024 [56]
Complementarity	The microbial partners express metabolic pathways that are lacking in, but imply fitness benefits for the host.	Fronk and Sachs 2022 [57]

Some of the special locations where animals cultivate mutualistic bacteria result from evolutionary modifications of cavities in which the secretion of exocrine glands (i.e., glands delivering secretions directly into body cavities or onto external surfaces [58]) accumulates [57, 59]. In the case of squids, the origin of the luminescent organ is related to the ink bladder and probably the accessory nidamental gland [60, 61]. The nidamental gland is also a cavity specialized in hosting symbiotic bacteria that protect the host’s eggs [62], with many similarities to the luminescent organ in the way of obtaining symbionts [63]. In fungus growing ants (subfamily *Myrmicinae*, tribe *Attini*), elaborate cuticular crypts of different depths and shapes for cultivation of antifungal-producing bacteria, are associated with exocrine glands opened in the cuticle [53]. These structures have been highly modified across the ants’ evolutionary history and such modifications are absent in other ant genera that do not cultivate fungus [53]. Exocrine glands in the integument are common places used by invertebrates and aquatic vertebrates [59] to compartmentalize symbionts, letting selective colonization and nurturing by evolving as *symbiotic organs* (Box 1 [57]). Among terrestrial vertebrates, only gut associated compartments are considered symbiotic organs (reviewed in [57]). However, symbiotic bacterial communities are commonly detected in their exocrine glands [64], which may, thus, have evolved as organs specialized in cultivating specific mutualistic bacteria. A prominent example of the exocrine glands supporting symbiosis with bacteria is the uropygial gland of birds.

The uropygial gland (also called preen gland) is the only exocrine gland with external opening in birds [65]. The uropygial secretion serves to waterproof and keep integrity of the plumage [66] and may function in scenarios of social communication, parasitism, and predation that may be partially mediated by mutualistic bacterial symbionts (reviewed in [64, 67]). As an exocrine gland, the uropygial gland may be an ideal place for the compartmentalization of beneficial bacteria [68]. Evidence of a diverse range of bird lineages hosting symbiotic bacteria within their uropygial gland is increasing [69–79]. Moreover, in several of those species, injecting antibiotics into the glands influenced the secretion composition and abundance of beneficial substances [69, 70, 72, 80–82]. This shows that symbiotic bacteria in the gland *complement* (Box 1) metabolic pathways of avian hosts. Thus, although the uropygial gland is not included in the list of organs that allow animals to form close association with mutualistic bacteria [45, 57], evidence from well-studied species suggests that it may indeed be a symbiotic organ in some avian groups.

**Fig. 1:**
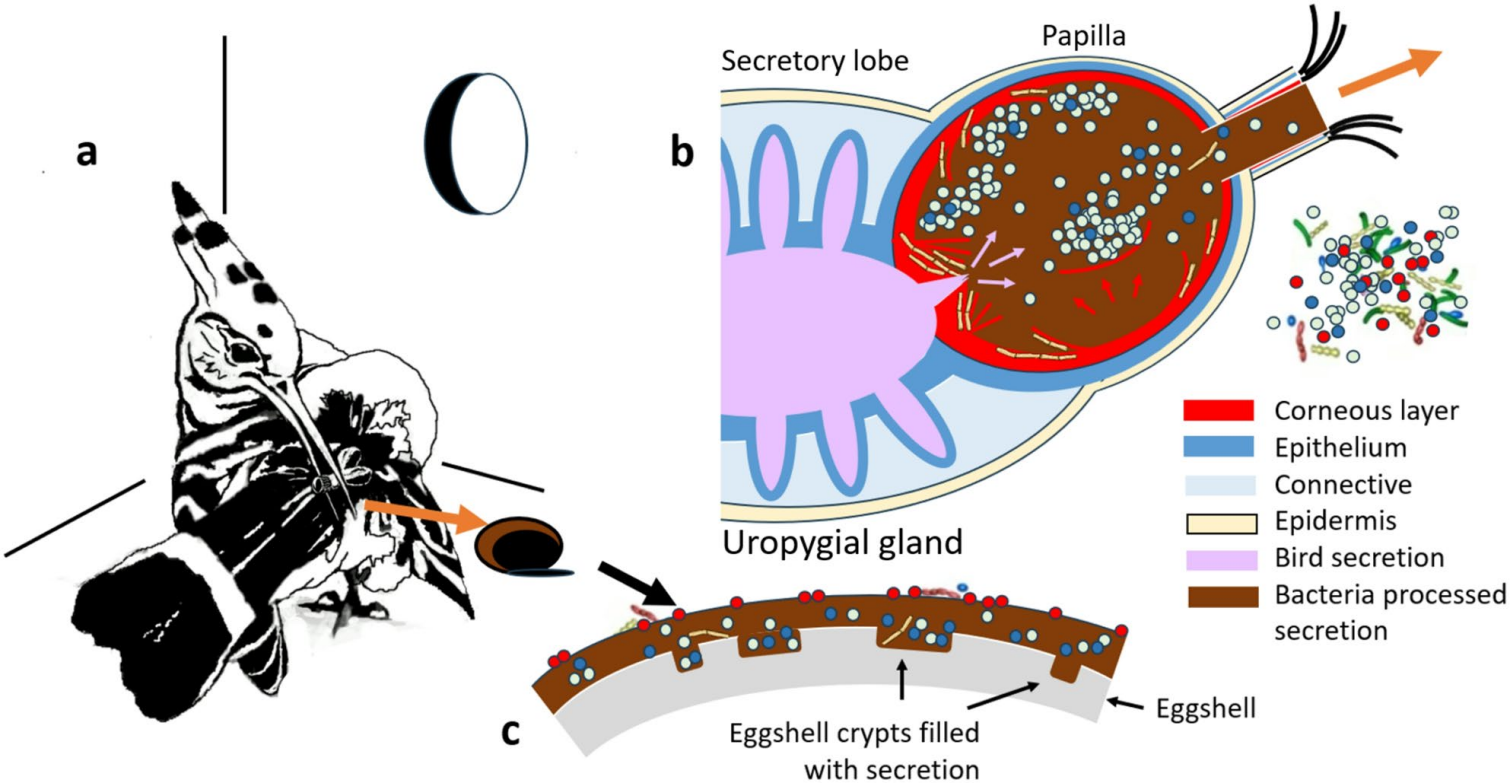
Structure and function of the hoopoe uropygial gland. (**a**) Nesting female hoopoe using the uropygial secretion to cover eggshell surface within the nest cavity. (**b**) Schematic representation of the section of the uropygial gland of a nesting hoopoe (breeding female or nestling). The papilla is full of dark secretion because of the metabolic activity of symbiotic bacteria living within it. Bacteria do not enter hoopoe tissues nor the cavities of the secretory lobe, since a thick keratin corneous layer compartmentalizes them within the papilla lumen. Symbionts living within the papilla are a selection of those available in the surrounding environment, and use the secretion and probably substances provided by desquamating keratin layers, whose inter-lamina spaces are inhabited only by long bacilli (probably some actinobacteria). (**c**) The surface of eggshells is covered in dark secretion with uropygial symbionts, but incorporate many other bacteria from the environment

There is strong evidence suggesting that the uropygial gland of Upupiformes is specialized in cultivating beneficial bacteria. The uropygial glands of the European hoopoe (*Upupa epops*) and the green woodhoopoe (*Phoeniculus purpureus*) are special in several aspects related to symbiosis with bacteria. First, their dark and odorous secretions include known chemicals from bacterial metabolites [81, 82]. Moreover, in the case of the European hoopoe, dark and odorous secretions only occur in reproducing females and nestlings (hereafter, both named as nesting individuals). These nesting individuals, in comparison with males and non-reproducing females, have bigger glands that harbor abundant and complex bacterial communities [82–84]. The existence within the same species of individuals/phases with and without the symbiosis, makes the hoopoe an ideal system to study adaptations caused by the interaction with the symbiotic bacteria. In the case of breeding females, the secretion is used to cover eggshells, which is facilitated by a special eggshell, full of shallow crypts, that help retaining it [85–87] (Fig. 1a, c). Interestingly, experimental antibiotic injection in the glands not only affects the chemical composition and color of the secretion, but also causes a gland-size reduction [84]. This suggests that symbionts mediate the seasonal changes in the gland morphology of the females of this species (i.e., there is a “phenotypically plastic” structure of the organ, Box 2 [57]). The uropygial gland and secretion of green woodhoopoes also contain functional bacterial communities but, in this case, neither the symbiotic association nor gland morphology change between sexes nor throughout the year [72, 81]. It is of interest that these interspecific differences in temporal associations with bacteria and uropygial gland morphology are related to specificity of some of their symbionts. In the case of the European hoopoe, high density of several generalist species (mainly *Enterococcus faecalis* and *Enterococcus faecium*) appeared in the secretion of nesting individuals [18, 73, 88, 89], while in the secretion of green woodhoopoe a new species (*Enterococcus phoeniculicola*) was described [72]. Notably, these two distantly related Upupiform species (included in two different families: Upupidae and Phoeniculidae, [90–92]) living in three different continents (Europe, Asia and Africa) have been found to harbor similar symbionts within their glands (species of the Genus* Enterococcus*, hereafter, enterococci) when studied with aerobic culturing methods [72, 89, 93]. For the European hoopoe, molecular analyses of the microbial communities indicate that a variety of taxa, other than enterococci, dominate the uropygial bacterial community [83, 94, 95] which is very different from those of other sympatric bird species [79]. Although the bacterial community of woodhoopoe glands has not been characterized with molecular methods, the similarity in the bacterial metabolites found in the secretions of the two Upupiform groups [81, 82] suggests that their symbiotic microbiota may share constituents, as would be expected for symbiotic microbiotas of specialized organs (“infection specificity”, Box 2, [57]).

In this study, we tested if the uropygial gland of the European hoopoe is a specialized organ for the selective cultivation of a particular microbiome and, thus, it meets the criteria assigned to typical symbiotic organs (Box 2 [57]). Briefly, we first analyze the specificity of host-bacterial association by comparing two populations breeding in two spatially independent countries (Switzerland and Spain) and with different migratory strategies [96, 97]. If the gland is acting to select a particular kind of symbiont, we expect to find congruence in the communities of uropygial secretions of both European hoopoe populations. Moreover, given that female hoopoes cover eggshells with uropygial secretion [85–87], but the eggshell community is more exposed to environmental influence [98], we expect to find higher inter-population variation and more diverse microbiomes on eggshells than for glands. Second, we study the anatomy and morphology of the uropygial gland of the European hoopoe. We expect to find morphological differences in glands of nesting and non-nesting hoopoes that point at the existence of compartmentalization favoring bacterial cultivation (Fig. 1b). Third, we study microscopically the organization of bacterial morphs within the gland of nesting hoopoes. We expect that different types of bacteria are associated with distinct within-gland spaces (Fig. 1b), implying selective compartmentalization/specificity of symbionts related to gland structure.

**Box 2:** Key features of symbiotic organs (modified from [57])

Feature	Function	Origin
Infection specificity	Symbiotic organs filter microbial partners.	a) Mechanical structures that spatially and/or temporally restrict access. b) Chemical attractants that help recruit symbionts. c) Antibiotic selectors that remove non-symbiotic strains.
Structure favoring compartmentalization	Structures that reduce symbiont interference with other host processes while creating microenvironments that optimize the services provided by symbionts.	The structure might be *canalized* (unaffected by symbiont colonization), or *phenotypically plastic* (promoted by symbiont colonization).
Ability to maintain beneficial partnerships	a) Within organ promotion of beneficial symbionts. b) Mediate transmission of best cooperators.	a) Host investment that generates nutritional support to beneficial symbionts or sanctioning of strains that disrupt the symbiotic organ or its function. b) Host physically transfers beneficial partners from symbiotic organs.

## Methods

### Study species

The European hoopoe (*Upupa epops*) inhabits open woods or open areas such as steppes, grasslands, pastures, semi-deserts, or field crops with scattered trees, walls or buildings providing holes for nesting, and fields with patches of bare ground for feeding [99–101]. Females lay one, two or rarely three clutches of 5–10 eggs over the breeding season, between February and July [102]. Often, they use the same nest hole through the years and between clutches in the same year [102, 103]. Incubation, performed only by females, lasts 16–17 days and starts with the first, second or third egg. Eggs laid after the start of incubation will hatch asynchronously at 24 h or even greater intervals [104–106], which causes a marked size hierarchy within the brood, frequently resulting in the death or cannibalism of last hatched nestlings [107–109]. The female stays within the nest, brooding chicks, at least for eight days after the start of hatching, and, for her whole stay since start of laying, all food for her and nestlings is provided by the male, which rarely enter the nest cavity [110].

### Study area

The study was carried out in two distant wild populations in Spain and Switzerland sampled during the 2010 and 2014 breeding seasons, respectively. The Spanish population is located in the Hoya de Guadix (37°24′08″N, 3°04′04″W), southern Spain, where hoopoes breed in nest-boxes placed in trees or buildings in crops, forests and gullies [102]. This population is a mixture of sedentary and migratory individuals spending the winter in Africa [96, 97]. The Swiss population is located on the plain of the Rhône, in Valais, southwestern Switzerland (46°16′10′′N, 7°7′29′′E). This area is a semi-open to open landscape with 700 nest-boxes installed mostly in agricultural sheds among fruit tree plantations and vineyards [111]. This population is integrated by long distance Palaeartic-African migrant individuals, which spend the non-breeding season in the Sahelian belt south of Sahara [96, 97, 112]. They typically return to the breeding grounds in March-May, start breeding in April, and the last nestlings usually fledge in August [103, 113].

### General methods

Nest-boxes were visited twice per week from mid-February to the end of July in Spain and once per week from mid-April to the end of July in Switzerland, to record laying date, clutch size and hatching date. All individuals were banded with metal numbered rings for individual recognition. Incubating females were caught 14 days after laying the first egg within the nest-box by hand, quickly sampled, and released again within the nest-box to minimize disturbance. Nestlings were sampled 20 days (± 1 day) after hatching of the first egg. All sampling was carried out with sterilized material and reagents, whether prepared at the laboratory or from the commercial supplier. For each capture, we wore new latex gloves cleaned with 70% ethanol for the whole process to reduce the risk of bacterial contamination. Before collecting uropygial secretion samples, we gently washed the circlet of feathers and skin surrounding the uropygial gland with a cotton swab dipped in ethanol to prevent contamination with external bacteria. Then, the secretion was extracted by inserting a sterile micropipette tip (1–10 ml Finpipette micropipette) into the opening while gently pressing the papilla (Fig. 1b), and transferred to a sterile microfuge tube. Bacterial samples from eggshells were collected by rubbing the entire surface of all eggs in the clutch with a sterile swab slightly wet with sterile phosphate buffer (PBS, Na_2_HPO_4_, 0.1 M and NaH_2_PO_4_ 0.1 M, pH 7.4) and individually stored in a sterile microfuge tube with 1.2 ml of the buffer solution [114]. Samples were kept cool (i.e., 3–4 °C) in the field until being stored at -20 °C in the lab for further molecular analyses.

### Bacterial diversity and taxonomic identification

Bacterial genomic DNA was extracted from uropygial secretions (10–20 µl per sample) using FavorPrep™ Blood Genomic DNA Extraction Kit (Favorgen Biotech). For eggshells, before DNA extraction, samples were sonicated in an ultrasonic bath (50/60 Hz) for 90 s to free cells from the cotton swab. Afterwards vials were centrifuged for 5 min at maximum speed. The pellets were treated with a lysozyme solution (10 mg/ml, 37 °C for 30 min) and extracted with the Chelex method [115]. The extracted DNA was eluted in sterile distilled water and stored at -20 °C until processing for microbiota analyses.

The bacterial communities were studied by two different approaches: 16S rRNA amplicon sequencing in the Illumina MiSeq platform [116] and ARISA (automated rRNA Intergenic Spacer Analysis [117]). We used ARISA in previous experimental studies exploring the dynamics of the bacterial community of the hoopoe secretion [87, 95, 118, 119], followed by studies based on 16S rRNA amplicon sequencing exploring the relationships of this community with nest odor and ectoparasitism risk [120]. Here, we used both ARISA and 16S rRNA amplicon sequencing of the v6-v8 region of 16S rRNA genes for microbiota characterization (see details of these protocols in Additional file 1). We used all available samples (*N* = 342) for ARISA, and a subset of them (*N* = 84) for 16S rRNA amplicon sequencing. The use of both methods allowed us to check for consistency of results between approaches in some analyses, and to study the most probable correspondence of ARISA peaks with taxa identified by 16S rRNA amplicon sequencing (Additional file 3). That exercise was used to discuss our results considering information about the hoopoe microbiota previously acquired with ARISA.

For ARISA analyses, we processed a total of 342 samples from 32 breeding attempts in Spain and 39 in Switzerland. Of these samples, 60 were eggshells (30 Spain, 30 Switzerland), 69 were female´ secretions (30 Spain, 39 Switzerland) and the remaining 213 were nestlings´ secretions (68 Spain, between 1 and 3 nestlings/brood; 145 Switzerland, between 1 and 7 nestlings/brood). For 16S rRNA amplicon sequencing, we used a subsample (*N* = 84) including 7 broods from Spain (2 nestlings per brood, 8 female secretions, eggshell samples from 6 clutches) and 15 broods from Switzerland (2 nestlings per brood, 14 female secretions, eggshell samples from 12 clutches). For both 16S rRNA amplicon sequencing and ARISA analyses, negative controls were run together with samples and in no case did they produce amplification. Detailed methods on sequence library processing of samples are included in Additional file 1.

The nomenclature used for the taxonomy assigned to 16S rRNA amplicon sequencing ASVs follows the database silva-138-99-tax, although some names have changed [121]. For example, we maintain *Firmicutes* for the actual phylum *Bacillota* or *Proteobacteria* for the actual phylum *Pseudomonadota*.

### Uropygial gland morphology and anatomy

Fresh uropygial glands of hoopoes found killed by predators in the field in the Spanish population (nine individuals), or found dead accidentally in our captive breeding population (eight individuals, [118]) were dissected and mounted for the study of internal anatomy and morphology. Glands were immediately fixed in 4% paraformaldehyde, washed in sterile distilled water, dehydrated in successive 50%, 80%, and 96% ethanol baths, rinsed in distilled water and stored in PBS at 4 °C until use.

Dehydrated glands were embedded in Embed 812 resin. Semi-thin sections of the samples were stained with hematoxylin-eosin and observed in an Olympus BX51 microscope and an Olympus DP72 camera (Olympus, Shinjuku, Tokyo, Japan) for histological study, or used for Fluorescence In Situ Hybridization (FISH) with fluorescently labelled probes for bacteria (see below).

Glands from 17 individuals were used for the microscopic study: four breeding females, four breeding males, three non-breeding females, three non-breeding males and three nestlings with functional glands (i.e., older than 8 days).

### Fluorescence in situ hybridization (FISH)

Semifine sections of the uropygial gland of a breeding hoopoe female were used to study the location of bacteria within the gland using FISH techniques. Samples were hybridized with Eub338 [122] labeled with cyanine 3 (Cy3) as a universal probe for bacterial RNA (Thermo Scientific, Ulm, Germany). As positive control for the presence of bacterial DNA, we incubated slides with the DNA intercalating agent Hoechst (Sigma-Aldricht, St. Louis, MO, USA). For a detailed explanation of the hybridization protocol followed, see Additional file 1. Using an Olympus BX51 fluorescence microscope and an Olympus DP72 camera, we obtained pictures at different magnifications of the gland around the papilla and secretory lobes. Pictures were taken through the 4’,6-diamidino-2-phenylindole (DAPI, blue), fluorescein-5-isothiocyanate (FITC, green-yellow), and tetramethylrhodamine-isothiocyanate (TRITC, red) filters, and superimposed with Photoshop.2024 when needed for a better visualization of the location within the gland.

### Statistical analyses

QIIME2 v2021.11 (Quantitative Insights In Microbial Ecology [123]), was used to generate alpha-diversity estimates for samples (number of ASVs, Berger-Harper dominance and Faith phylogenetic distance [124]), and beta-diversity matrices of distances among samples (weighted and unweighted UniFrac distances [125]). We have used UniFrac distances of rarefied samples (Additional file 1) as an analytical approach to compare composition of communities [126, 127]. Comparisons among types of samples were performed with Primer 7.0.13 (PRIMER-e) and Statistica TIBCO 2023.

Comparison of alpha-diversity indices was performed using General Linear Mixed Models (GLMM), with sample type (nestling secretion, female secretion and eggshells) and population (Spain, Switzerland) as fixed factors, and nest identity (nested within population) as random factor. A factorial design including the interaction between sample type and population explored whether population affected differences among sample types, and when non-significant, the interaction was excluded from the final model.

The beta-diversity of bacterial communities was compared among sample types and populations (Spanish and Swiss) using the same design described above, by means of PERMANOVA with 9999 permutations [128]. Distance matrices were calculated using unweighted and weighted UniFrac distances generated with QIIME2 for 16S rRNA amplicon sequencing at the ASV level, and Jaccard distances for ARISA ITSs.

Classical multidimensional scaling analysis (MDS after bootstrap analyses with 50 repeats per group) was used to graphically visualize bacterial community composition of the samples in multidimensional space. This technique represents the communities on a plot with canonical axes, where the proximity between communities shows their underlying similarity [129].

To explore whether the bacterial communities of the two populations (Spain and Switzerland) are congruent, we analyzed the relationship between prevalence in Switzerland (dependent variable) and Spain (continuous explanatory variable) for ASVs in Generalized Linear Models (GLZ) with a Poisson distribution. Sample type and its interaction with prevalence in Spain were included as fixed factors. To elucidate the predicted greater dissimilarity between populations for eggshells than for secretions, the models were repeated including only the two types of secretion samples.

All samples were used to generate the abundance-occupancy plot of ASVs expected under a neutral model for secretions and eggshells [130]. This allowed us to evaluate the position of ASVs out of the confidence interval of the neutral model, suggesting positive or negative selection [131]. Additionally, we determined the members of the core microbiome, i.e., consistent ASVs of our data set that can be hypothesized to reflect underlying functional relationships with the host [131, 132]. For this approach we calculated the consistency index for each taxon found in secretions using the available script for spatial study designs provided by Shade and Stopnisek (2019) [131]. As cut-off criterion we used the last taxon (ranked by the consistency index) increasing more than 2% the beta diversity of the core [131].

## Results

### Influence of sample type and population on microbiome composition

**Fig. 2:**
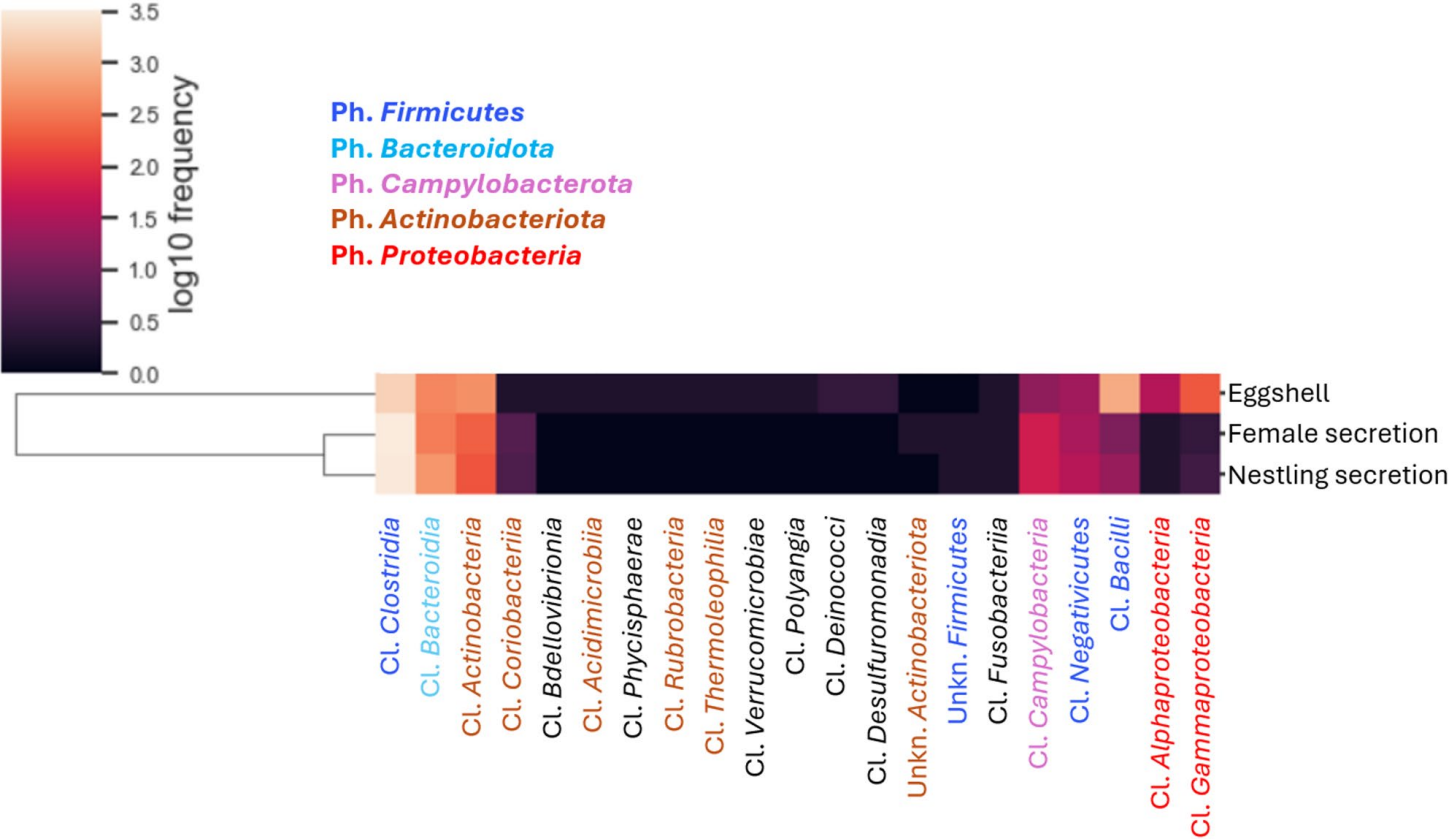
Heatmap showing differences in the relative abundances of bacteria at the Class level among three sample types obtained from nesting hoopoes. Nestling and female uropygial gland secretions clustered together and showed similar profiles. Eggshells were differentially enriched in both *Alpha*- and *Gammaproteobacteria*, as well as *Bacilli*. Bacterial classes label colors refer to their taxonomic classification into the phyla listed in the figure (the seven classes written in black are from seven different minoritarian phyla exclusive of eggshells (except *Fusobacteriia*), whose names are not included in the legend)

The 16S rRNA amplicon sequencing of the 85 hoopoe samples produced 1,922,850 reads (mean(SD) = 22,621.76(7,226.21)) classified into 677 ASVs. After rarefaction to the minimum sampling depth (3,864 reads, see Additional file 1), the number of ASVs was reduced to 661 for the 84 samples retained. Taxonomy assignment detected 12 bacterial phyla, with a much higher relative abundance of *Firmicutes* (78.4% of reads) followed by *Bacteroidota* (12.2%), *Actinobacteriota* (6.7%), *Proteobacteria* (1.3%), and *Campylobacterota* (1.3%). The remaining seven phyla except *Fusobacteriota* were exclusively found on eggshells (classes written in black in Fig. 2). The Phyla *Proteobacteria* and *Actinobacteriota* were significantly more frequent in eggshells samples (Fig. 2, Kruskal-Wallis tests on relative abundance, H(2, *N* = 84) = 49.1, *p* < 0.001; H(2, *N* = 84) = 23.2, *p* < 0.001, respectively), while Ph. *Firmicutes* reached higher values in secretions (Fig. 2, Kruskal-Wallis test, H(2, *N* = 84) = 11.97, *p* < 0.005), without significant differences between nestlings and female secretions (Additional file 2, Table S1). Within the Ph. *Firmicutes*, the relative abundance of Cl. *Bacilli* was much higher in eggshells, whereas Cl. *Clostridia* was dominant in secretion samples (Fig. 2).

**Fig. 3:**
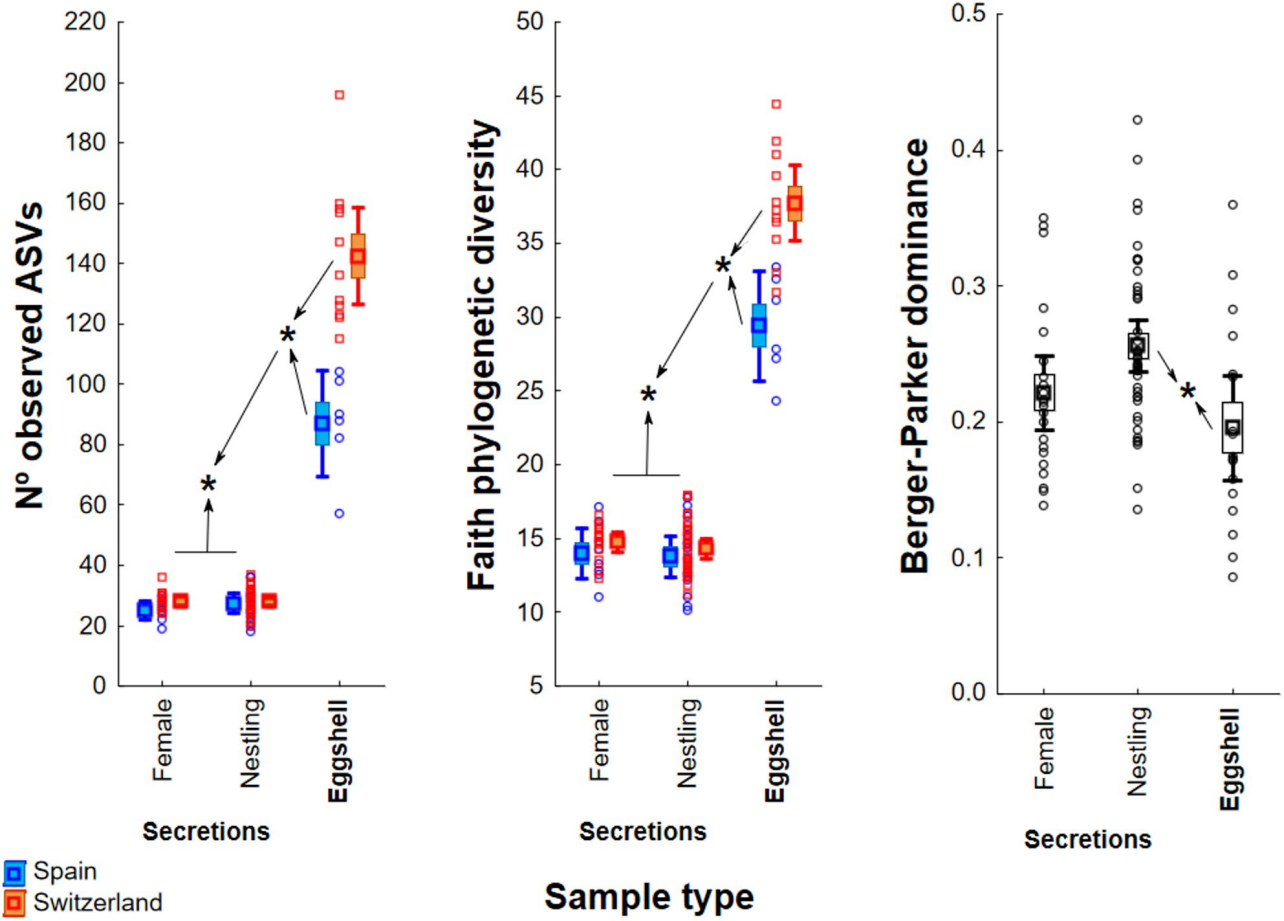
Differences among the three sample types and between populations in three alpha-diversity estimates (boxplots show Mean ± 95% CI and SE; points are raw data). For Nº of ASVs and Faith phylogenetic diversity there was a significant interaction between population and sample type, while population had no effect on Berger-Parker dominance (Additional file 2: Table S2). Asterisks mark significant differences in post-hoc Tukey tests between groups connected by arrows (*p* < 0.05)

The higher bacterial diversity on eggshells was also evident at the ASV level. Eggshell samples presented significantly higher alpha-diversity than secretion samples for number of ASVs and Faith phylogenetic diversity, which did not differ between nestling and female secretions (Fig. 3, Additional file 2: Table S2). The Berger-Parker dominance index was significantly higher for nestling secretions than for eggshells, with an intermediate mean value in female secretions (Fig. 3, Additional file 2: Table S2). The number of ARISA ITSs per sample showed a different pattern, with significantly higher values for Spanish than Swiss secretions and the opposite for eggshells (Additional file 2: Fig. S1).

**Fig. 4:**
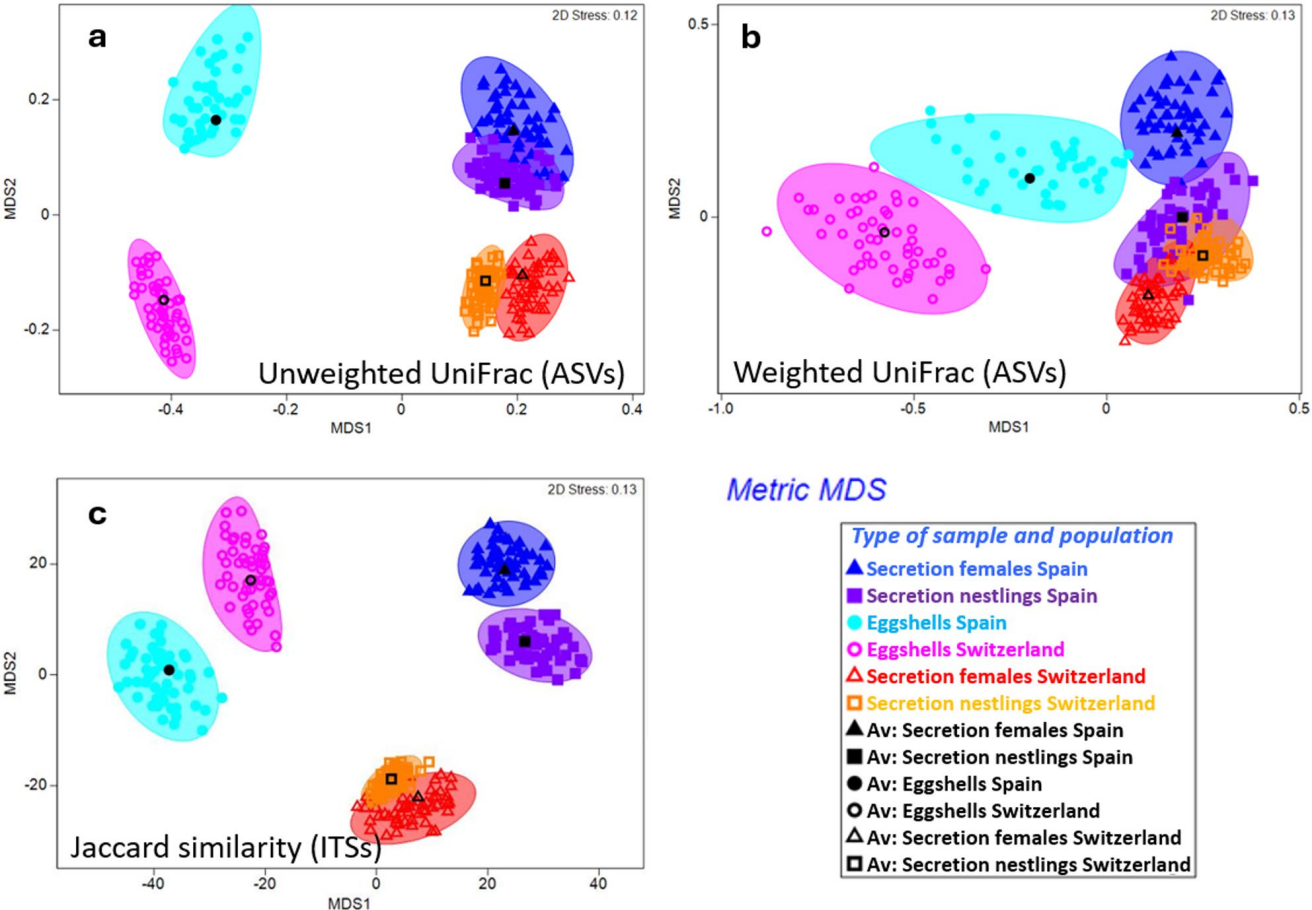
Differences in the composition of the hoopoe bacterial communities present in the two types of uropygial secretions (females and nestlings) and eggshells, for the Spanish and Swiss populations. Graphs show the average positions of communities in a metric multidimensional space, after bootstrap analyses with 50 repeats per group. (**a**) Comparison based on unweighted UniFrac distances at ASV level, (**b**) Comparison based on weighted UniFrac distances at ASV level (**c**) Comparison based on the 63 most prevalent ARISA ITSs based on Jaccard similarity. Black symbols mark the average positions obtained in the bootstrap analyses and shaded areas the 95% confidence surface of such means. See Permanova Models for comparisons in Additional file 2: Table S3

Using beta-diversity estimates to study community composition, we found greater distances within populations between eggshells and secretions than between nestling and female secretions, both with unweighted UniFrac distances for 16S rRNA amplicon sequencing ASVs and with ARISA ITS identities (Fig. 4; Table 1, Additional file 2: Table S3 for ARISA). This pattern was less clear using weighted UniFrac distances for Spain, without any significant pairwise comparison (Table 1). The differences between populations were greater for eggshells than for both secretion types when using unweighted UniFrac distances (Fig. 4a, see average distances in Table 1). A similar trend but much less pronounced was obtained for weighted UniFrac distances and similarity in ARISA ITS composition (Fig. 4b-c; Table 1 and Additional file 2: Table S3 for ARISA). The beta dispersion around centroids were not significantly different between populations for any sample-type x beta diversity index, except for Jaccard distances in nestling secretions (Permdisp results in Table 1 and Additional file 2: Table S3). However, in most cases, the comparisons of beta dispersions were significant for nestling and female secretions within countries, coinciding with non-significantly different or less distant communities of secretions when compared with Permanovas (Table 1 and Additional file 2: Table S3).

**Table 1:** Results of Permanova analyses comparing the composition of the hoopoe bacterial communities present in the two types of uropygial secretions (females and nestlings) and eggshells, for the Spanish and Swiss populations

Permanova											
a) Unweighted UniFrac						b) Weighted UniFrac					
Whole model						Whole model					
***Factor***		***Nested in***	***df***	***PseudoF***	***p***	***Factor***		***Nested in***	***df***	***PseudoF***	***p***
1) Population	Fixed		1	2.15	**0.0001**	1) Population	Fixed		1	2.33	**0.02**
2) Sample type	Fixed		2	18.38	**0.0001**	2) Sample type	Fixed		2	9.07	**0.0001**
3) Nest	Random	Populat.	22	4.68	**0.0001**	3) Nest	Random	Populat.	22	3.57	**0.0001**
1 × 2	Fixed		2	2.44	**0.0001**	1 × 2	Fixed		2	2.32	**0.02**
2 × 3	Random		34	1.71	**0.0001**	2 × 3	Random		34	1.55	**0.03**
Res.			22			Res.			22		
**Pair-Wise tests (1 × 2)**						**Pair-Wise tests (1 × 2)**					
**Factor**	**Within**		**Distance**	**t**	**p**	**Factor**	**Within**		**Distance**	**t**	***p***
Population	Eggshells		0.59	1.69	**0.0003**	Population	Eggshells		0.88	1.39	0.098
Population	Nestlings’ secretions		0.51	1.12	0.141	Population	Nestlings’ secretions		0.70	0.89	0.572
Population	Females’ secretions		0.47	1.57	**0.002**	Population	Females’ secretions		0.67	1.97	**0.002**
**Factor**	**Within Spain**		**Distance**	**t**	p	**Factor**	**Within Spain**		**Distance**	**t**	**p**
Sample type	Eggshells-Fem. Secret.		0.75	2.88	*0.02	Sample type	Eggshells-Fem. Secret.		0.73	1.14	0.320
Sample type	Eggshells-Nestl. Secret.		0.78	2.59	**0.02**	Sample type	Eggshells-Nestl. Secret.		0.77	1.51	0.159
Sample type	Fem.-Nestl. Secretions		0.47	1.29	*0.174	Sample type	Fem.-Nestl. Secretions		0.64	1.64	0.109
**Factor**	**Within Switzerland**		**Distance**	**t**	**p**	**Factor**	**Within Switzerland**		**Distance**	**t**	**p**
Sample type	Eggshells-Fem. Secret.		0.76	4.54	*0.0001	Sample type	Eggshells-Fem. Secret.		1.06	3.43	*0.001
Sample type	Eggshells-Nestl. Secret.		0.76	4.67	**0.0001**	Sample type	Eggshells-Nestl. Secret.		0.99	3.97	**0.0001**
Sample type	Fem.-Nestl. Secretions		0.47	1.29	*0.01	Sample type	Fem.-Nestl. Secretions		0.66	2.05	*0.02

The matrix of distances among samples used is based on: (a) Unweighted UniFrac and (b) Weighted UniFrac distances, calculated with the profiles of ASVs obtained with 16S rRNA amplicon sequencing rarified to 3864 sequences per sample. The whole model as well as the pair-wise tests comparing the same type of sample between populations (factor 1 × 2 within type of sample) are presented. Asterisks on pair-wise *p* values, mark when the comparison of beta dispersions using a Permdisp analysis resulted significant (*p* < 0.05)

**Fig. 5:**
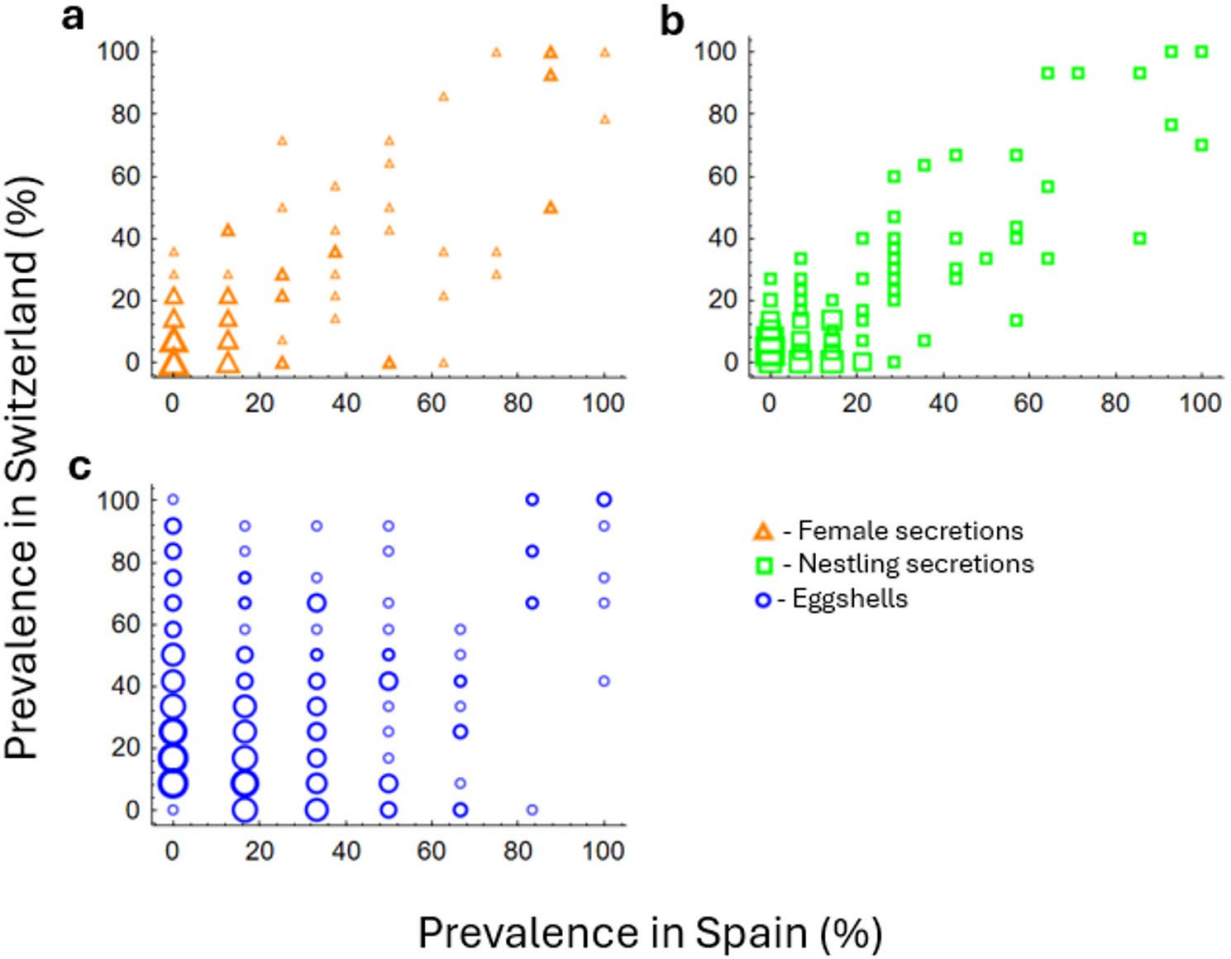
Relationships between the prevalences in Spain and Switzerland for 16S rRNA amplicon sequencing ASVs in each of three sample types: (**a**) female secretions, (**b**) nestling secretions and **c**) eggshells. Number of ASVs per point is represented on a log scale

Prevalence of bacterial ASVs associated positively between Spanish and Swiss populations (Fig. 5), while the strength of the association depended on the considered type of sample (GLZ, interaction term, 16S rRNA amplicon sequencing ASVs: Wald = 1757.194, *p* < 0.001, Fig. 5). The significant interaction with type of sample for ASVs was mainly due to differences between eggshells and both types of uropygial secretions, since it became non-significant when eggshells were excluded (Wald = 1.325, *p* = 0.250, Fig. 5). Given the great similarity in the profiles of female and nestling uropygial secretions within populations (Figs. 2, 3, 4 and 5; Table 1), we have considered them together for subsequent analyses.

**Fig. 6:**
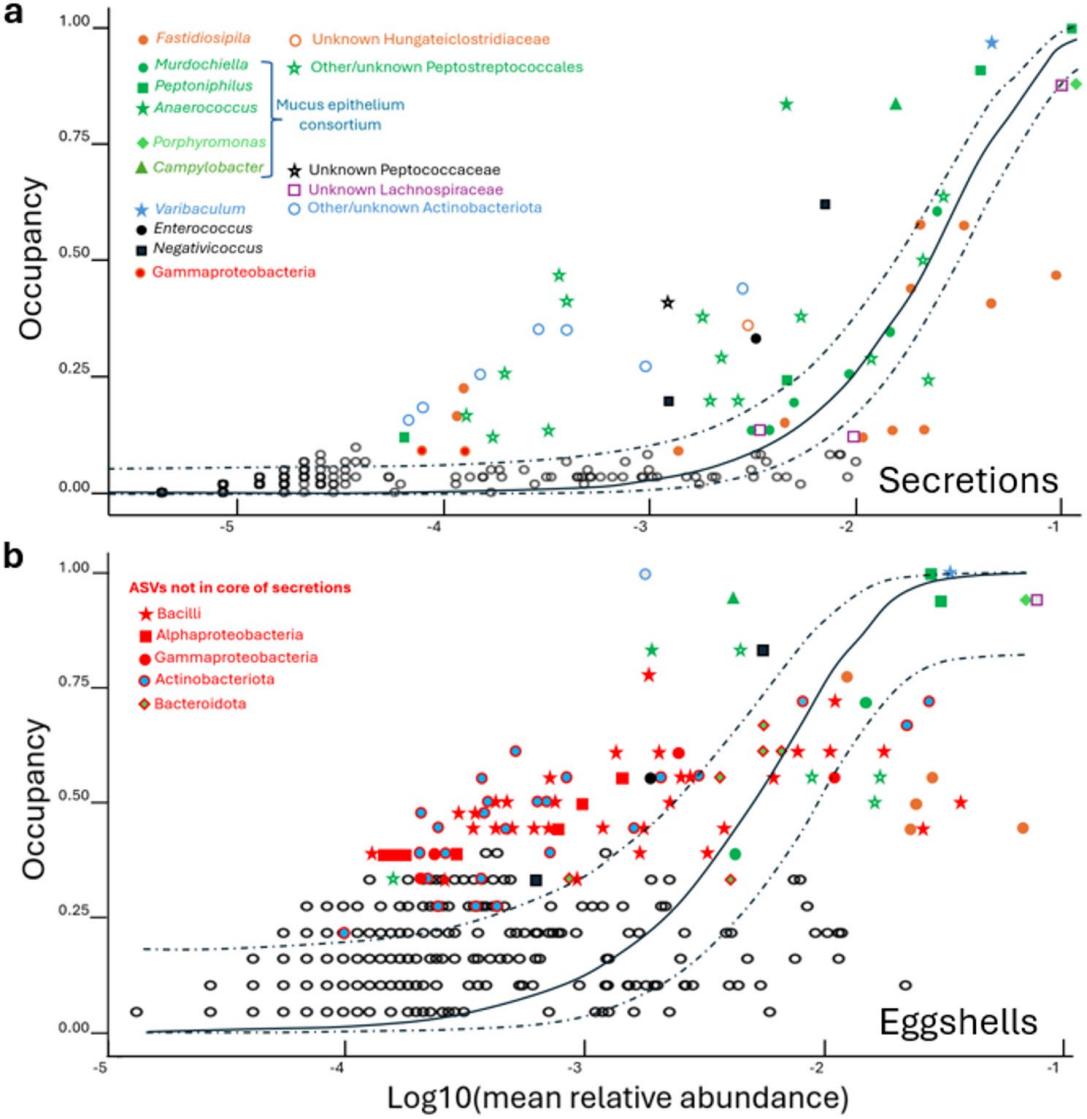
Contribution of the different ASVs found in hoopoe uropygial secretions to the similarity in the composition of bacterial communities among samples. (**a**) Fit of the neutral model to the relationship between abundance and occupancy for the ASVs found in uropygial secretions of both Spanish and Swiss hoopoes. The solid line represents the expected relationship of the neutral model, and dashed lines are 95% CI around the neutral model. Points represent different ASVs, the 59 included in the core are determined taxonomically as indicated by color and shape in the legend. Those out of the CI interval are more (above) or less (below) prevalent than expected by their abundance under a neutral model. (**b**) Fit of the neutral model for eggshells in both populations. ASVs not present in the core microbiome of secretions are marked with red symbols and their taxonomic affiliation indicated in the legend. The ASVs shared with secretions maintain the same symbols from subfigure a

The taxonomic affiliation of ASVs of the core microbiomes showed that a few from four phyla dominated all communities (Fig. 6, only analyzed for 16S rRNA amplicon sequencing, the correspondence of ARISA ITSs with identified ASVs is explored in Additional file 3). The same ASVs with the highest prevalence in secretions: one *Porphyromonas* (Ph. *Bacteroidota*), one *Varibaculum* (Ph. *Actinobacteriota*), one *Campylobacter* (Ph. *Campylobacterota*), and nine AVSs from phylum *Firmicutes* including one *Negativicoccus*, one *Lachnospiraceae*, two *Fastidiosipila* and five *Peptostreptococcales* (one *Anaerococcus*, two *Peptoniphilus*, one *Murdochiella* and other unidentified), also occupied the highest rank positions in the core microbiome of eggshells (Fig. 6). However, the remaining part of the core was very different between secretions and eggshells. The eggshell community was enriched with numerous ASVs of *Bacilli* (Ph. *Firmicutes*), *Proteobacteria*, *Bacteroidota* and groups of the Phylum *Actinobacteriota* not present in the core of secretions. On the other hand, the composition of the core microbiome of secretions was more consistent in its whole range, with a great proportion of potentially redundant ASVs. In many cases, the core ASVs of secretions were variants of the same genus or closely related genera, mainly within *Firmicutes.* For instance, 13 ASVs were *Fastidiosipila* variants (Ph. *Firmicutes*, O. *Clostridia*), five were *Murdochiella* (Ph. *Firmicutes*, O. *Peptostreptococcales)*, the genus *Peptoniphilus* with the most prevalent (found in 100% of secretions) and abundant ASV, presented 4 variants (also O. *Peptostreptococcales*), and there were 15 additional *Peptostreptococcales* ASVs (two *Helcococcus* and 13 unidentified, Fig. 6a). In many cases, variants of the same genus were negatively correlated or even incompatible (results not shown) suggesting they are alternative symbionts filling the same niche. Two ITSs known to depend on vertical transmission from female glands to nestlings corresponded to genera of the order *Peptostreptococcales* (*Helcococcus* and *Peptoniphilus*) included in the core of secretions (Additional file 3: Table S2). Prevalence of most of the 59 ASVs of the core of secretions (32 out of the 59) was higher than expected by their relative abundances under a neutral model (Fig. 6a). When the core microbiomes were studied separately by population, a high proportion (76.8%) of the total 82 ASVs included in secretions were shared, while only 25% of 179 core ASVs from eggshells were shared between the Spanish and Swiss populations (Chi-Square test: ꭓ2 = 153.37, *p* < 0.0001).

### Specializations of uropygial gland in nesting individuals

**Fig. 7:**
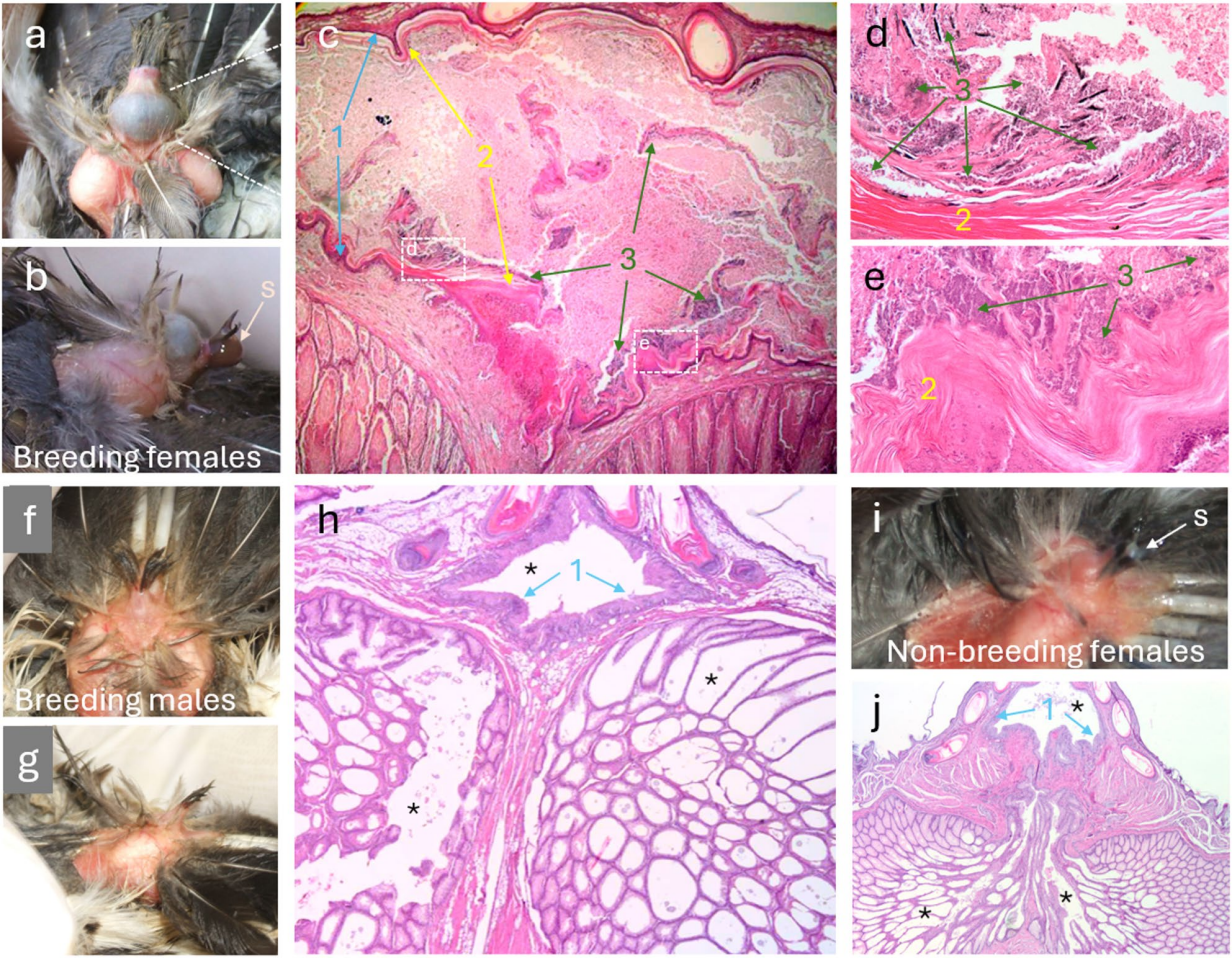
Morphology and anatomy of the uropygial gland of hoopoe breeding females (**a-e**), breeding males (**f-h**) and non-breeding females (**i-j**). Images **c**,** d**,** e**,** h** and **j** are hematoxylin-eosin-stained sections of glands, showing the internal anatomy of the papilla and its connection with the secretory lobes (the approximate spatial correspondence is indicated with dashed lines in image **a**). Images **d** and **e** show details of the internal wall of the papilla at the sites indicated in image **c**. Numbers mark: **1** basal and proliferative layers of the epithelium of the papilla wall, **2** corneous layers of the papilla wall, **3** masses of bacteria (thin grains violet-stained). The **S** in **b** and **i** marks a drop of secretion at the tip of the papilla. Asterisks mark empty spaces at the papilla and secretory lobes. Images **c**, **h** and **j** 40x, images **d** and **e** 400x

The external morphology and the internal anatomy and histology of nesting individuals (nestlings and breeding females) greatly differed from those of males and non-nesting females (Fig. 7). Regarding external morphology, the uropygial gland of nesting individuals shows a more voluminous papilla than that of non-nesting hoopoes (cf. Figure 7a, i). Because the stored secretion is dark in nesting individuals, the color can be seen through the skin, resulting in darker papilla compared to the secretory elements (lobules) (Fig. 7a-b). This is not true for non-nesting adults (Fig. 7f-g, i) whose secretions are white (Fig. 7i).

**Fig. 8:**
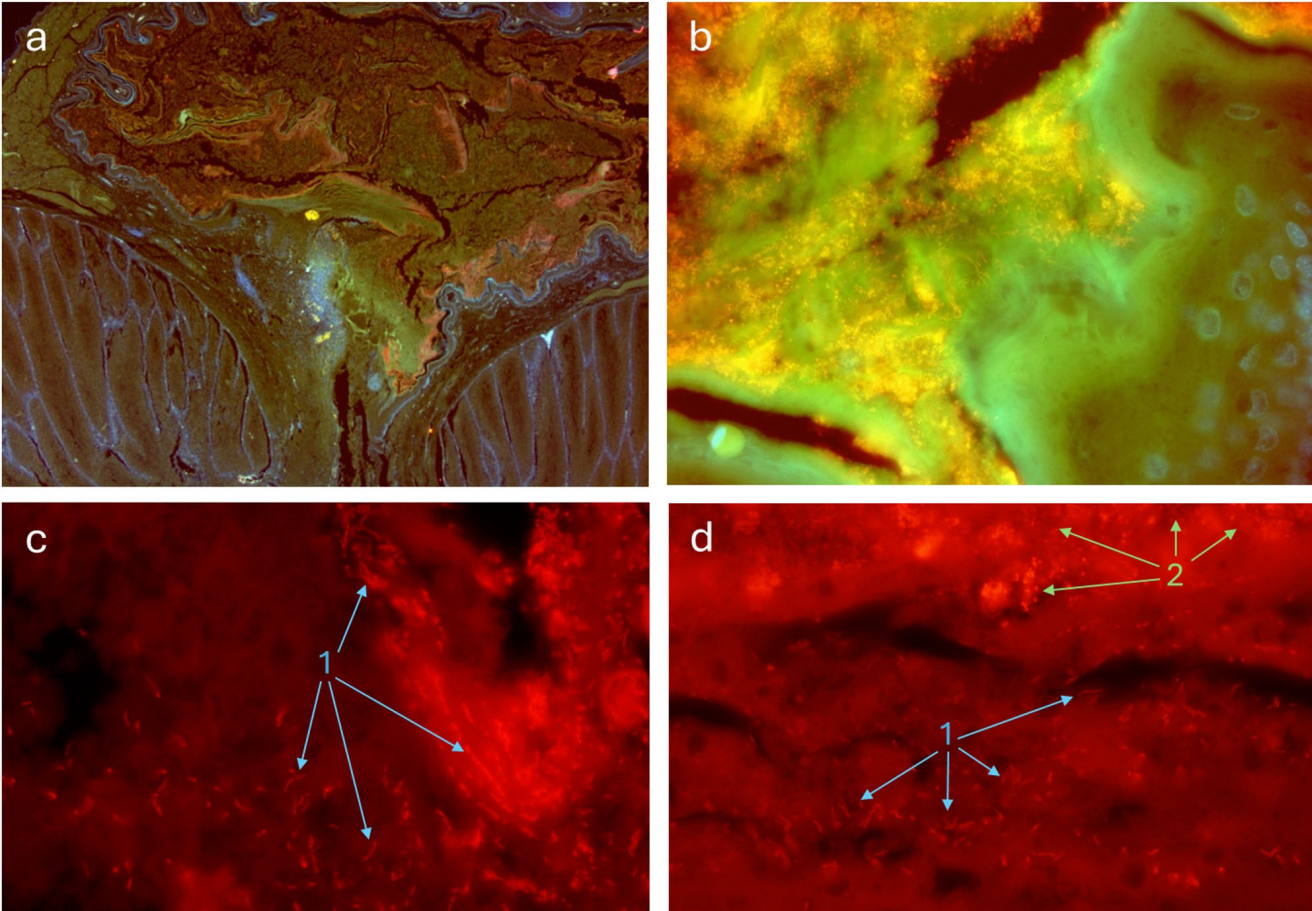
Fluorescence microscopical images of uropygial glands of hoopoe breeding females showing the localization of symbiotic bacteria within the papilla. Bacteria are fluorescently labelled in red with Cy3-EUB338 universal bacterial probe. In blue, nucleic acids of the bird cells nuclei stained with Hoescht. Images **a** and **b** are superpositions of the red, blue and green filters pictures so that the labelled bacteria look pale orange. (**a**) General view of the same section of the papilla presented in Fig. 6c. The whole content of the papilla is full of fluorescently marked bacteria, but the highest densities are located close to the papilla walls and detached corneous layers. (**b**) Detail of dense masses of bacteria filling all spaces among detached corneous layers in the papilla lumen. The bacteria do not surpass the thick integer keratinized surface of this layer. (**c-d**) High magnification of the interface between the papilla lumen and the thick corneous layer of the papilla wall. Bacteria of different morphologies are located within the detaching layers (chains of long bacilli indicated with **1**) and in the secretion filling the open areas (masses of cocci, indicated with **2**). Image **a** 40x, image **b** 600x, image **c** and **d** 1000x

We found interesting locations of symbiotic bacteria within the glands’ internal structure of nesting individuals (see female glands in Fig. 7 and Additional file 2: Fig. S2, nestling glands only in Additional file 2: Fig. S2). First, within the papilla, bacteria concentrate close to the internal walls or around corneous lamina disaggregated from it (see green arrows in Fig. 7c-d and specific fluorescent stained bacteria in Fig. 8a). Second, the internal wall of the papilla is separated from its lumen by a continuous keratin multi-stratified layer covering the epithelium, which is engrossed and folded around the connection between the papilla and the ducts coming from secretory lobes (yellow arrows in Fig. 7c). The magnification of that layer shows dense concentrations of bacteria, especially in the spaces left by disaggregating laminas (Fig. 7c-e). Such corneous layers are not present neither in males (seven individuals examined) nor in non-breeding female glands (three individuals examined), where the basal and proliferative layers of the epithelium appear in direct contact with the lumen of the papilla (blue arrows in Fig. 7h and j). Finally, an apparent additional difference between the images of the glands of the two types of individuals is the aspect of the material filling the secretory tubules, lobe spaces and papilla. Preparations of non-breeding adults normally show empty papillae and lobe spaces (asterisks in Fig. 7h and j), as well as abundant oil droplets inside cells in the intermedium-transitium layers of the epithelium of secretory tubules (Additional file 2: Fig. S3c, S3f). On the contrary, glands of nesting individuals stay filled with material after fixation, both in the papilla lumen and within the lobe spaces (Fig. 7c, Additional file 2: Fig. S2a, c). Moreover, the cells within the secretory tissues of tubules lack oil droplets in both breeding females and nestlings (Additional file 2: Fig. S2d-e).

### Compartmentalization and sorted organization of bacteria

The observation of fluorescently labeled bacteria confirmed compartmentalization and some level of organization of different types of symbionts within the papilla of the gland (Fig. 8). The corneous layer compartmentalizes the symbionts strictly in the papilla, with dense concentrations on the lumen side of the epithelium, but no bacteria in the lobes or live host tissues behind the corneum (Fig. 8a-b). Moreover, while open spaces are full of cocci (Fig. 8b), the engrossed areas of corneous material around the base of the papilla appear as loose laminas leaving narrow spaces occupied only by chains of long bacilli (Fig. 8c-d).

## Discussion

We have found that, in comparison with the bacterial community of the eggshell surface, the microbiota hosted within the uropygial gland of nesting hoopoes is very similar across populations. Moreover, most of its core components are more prevalent than expected by neutral processes, possibly implying some kind of filtering or promotion explaining its maintained composition. Indeed, the microscopic study of those glands showed that the bacteria are strictly compartmentalized within the papilla and concentrated on locations where the nesting hoopoes provide their special secretion and engrossed keratin layers, suggesting the possibility of host control. Moreover, morphologically distinct bacteria are associated with disaggregating keratin laminae. Such structures and organization are not present in the glands of males and non-breeding females, whose secretions differ from those of nesting individuals in bacteriological, physical and chemical properties [82–84]. Previous studies demonstrated that these symbiotic bacteria benefit their nesting hoopoe hosts [18, 73, 82, 85, 88, 93, 120, 133], suggesting that the glands of nesting hoopoes have evolved as specialized organs for growing a particular beneficial microbiome.

A first condition that the uropygial gland of hoopoes should fulfill to be considered as a symbiotic organ is that, independently of the environmental conditions, it should harbor consistent bacterial communities among populations (specificity, Box 2 [57]). Our results support that prediction. First, and although samples from the two populations were taken in different years, the communities of secretions were more similar between populations than those of eggshells. Second, the prevalence of bacterial ASVs in different populations were more consistent when considering uropygial secretions than eggshells bacterial communities. Moreover, alpha diversity was significantly higher in eggshell than in secretion microbiomes, and the prevalence of ASVs of the core microbiome was higher than expected by neutral processes. Taken together, these results suggest facilitation of the acquisition and growth of the particular community found in the uropygial gland of hoopoes.

The specificity of the core microbiome was even clearer when looking at the taxonomy of the detected bacterial ASVs of the uropygial secretion of nesting hoopoes in Spain and Switzerland. Bacterial ASVs that differed between both populations were mostly variants of the same genera. The taxonomic spectrum of this core microbiome is therefore very narrow. Moreover, seven ASVs were almost omnipresent in secretions from both populations, suggesting they are key constituents of this microbiome. We found that these seven ASVs were also present in the very different core microbiome of eggshells, suggesting their arrival after female application of secretions to eggs [85, 87, 95]. Five of them are typical components of the “mucosal consortium” (name given in [134]) characteristic of some regions of the mammalian intestine. Another 15 variants in the core were also unknown *Peptostreptococcales-Tissierellales* and therefore closely related to members of the consortium. These genera, and other main components of the core microbiome of glands (*Lachnospiraceae*,* Negativicoccus*,* Varibaculum*,* Fastidiosipila*,* Enterococcus*) are common constituents of the gut microbiome of healthy mammals and/or birds, many of them with known key functions in regulation of immune function or complementing metabolic routes [134–142]. If the same functions are demonstrated for the species/variants living in hoopoe uropygial glands, this would imply that these glands are recruiting a consortium of commensal gut bacteria into an organ not involved in food processing, but in bird-secretion processing instead.

A second property of symbiotic organs is the existence of specialized structures compartmentalizing bacteria (Box 2 [57]). The hoopoe is the only bird species in which, the conduits transporting the secretion from the two lobes of the uropygial gland to the opening form a common, wide cavity in the papilla, not divided by longitudinal septa and opening in only one orifice [65, 143]. Even the specialized papillae present in passerines, with valves preventing the backflow of secretion to the lobes, is divided by a septum and open in two orifices [143]. Inside that cavity is where nesting hoopoes store a dense, dark, stinky secretion [144] full of bacteria [84]. In the present study, we have shown that the stratum corneum of the epithelium lining the interior of the papilla in nesting hoopoes is constituted by a thick layer of long cornified keratin laminae similar to the structure of bird skin in unfeathered and exposed areas [145]. There is a striking difference with the papilla of males and non-breeding females, in which the corneous layer lacks such laminae. Instead, corneocyte apoptosis apparently happens in loose fragmented cell debris, similar to the non-cornified squamous epithelium typical of the internal ducts or the cavities of the papilla of other birds, such as passerines or pigeons [65]. These particularities of the papilla of nesting hoopoes may be an adaptation to compartmentalize that cavity, allowing its use as a reactor for cultivating bacteria while reducing the risk of invasion of hoopoe tissues. Indeed, corneous layers of epithelia are known barriers protecting against bacteria penetrating tissues at animal surfaces [146], as mucus layers do in internal organs such as the digestive tract [147–149].

In accordance with the existence of a compartmentalized cavity for bacterial cultivation, the FISH images showed that the papilla is full of bacteria, but they do not enter the lobes or penetrate the papilla wall. Moreover, bacteria appeared in association with disaggregated corneous laminae, which may serve as a source of nutrients for enhancing the growth of beneficial symbionts (third property of symbiotic organs, Box 2 [57]). It is known that the stratum corneum of the avian skin allows the transport of substances to the surface. This occurs by means of lipids of multigranular bodies in droplets freed in the intercellular spaces [150], which, for example, are used by pitohuis to channel defensive toxic alkaloids to the skin surface [151]. This functionality of the corneous layers could serve to provide a variety of substances to the epithelium surface [152] and drive the composition of the microbiome, as shown for the animal skin [146, 153]. Magnification of the corneous layers of the papilla in nesting hoopoes showed that only a particular type of long bacilli occupied spaces between those laminae. This suggests specialization in the use of nutrients of such location, either from the droplets present among laminae or from the very keratin of laminae (Fig. 1b).

The secretion produced by hoopoe tissues before reaching the papilla (i.e., in secretory lobes of the gland) was different in chemical nature between nesting and non-nesting hoopoes. This was shown by the dissolution of the content in cavities and ducts with non-polar solvents (i.e., ethanol used for fixing glands, e.g., [154]), only in non-nesting individuals. This suggests that, in the secretions produced by nesting individuals, the lipidic fraction is not so important as in non-breeding ones, as well as in most bird species [65]. In accordance with this possibility, only in these non-nesting individuals, the cells in the intermediate layers of secretory tubules of the gland showed lipoid-bearing spheres filling the cytoplasm (as it is common in most birds, e.g., [154, 155]). These results indicate that the kind of substances produced in the secretory lobes of hoopoe uropygial glands change substantially between the non-nesting and the nesting phase. Among other mechanisms, hosts regulate the dynamics of bacterial assemblages by secretions providing particular nutrients [44], or substances that enhance (or prevent) recruiting particular symbionts [49]. The shift in secreted substances detected in hoopoes may allow them to control the community hosted (Fig. 1b).

Symbiotic communities can be the result of an appropriate selection of symbionts by a combination of selective feeding of beneficial colonizers and vertical transmission [156]. Behavioral traits of hoopoes favoring vertical transmission of beneficial bacteria [87] or from nest materials from previous reproduction [157] could also explain the detected specificity of the bacterial community of the uropygial secretion of nesting hoopoes. Evidence of vertical transmission comes from correlational and experimental studies [118, 119, 158] in which fingerprinting techniques allowed to conclude that ITSs of sizes 346 and 466 were those most likely transferred from mothers to offspring [118]. Here, our results suggest that these ITSs correspond with the genera *Peptoniphilus* and *Helcococcus*. Further, two ITSs identified here as *Peptoniphilus* (sizes 346 and 566) were negatively related to the abundance of enterococci within the gland [118], and enterococci are responsible for the production of potent bacteriocins within hoopoe glands with beneficial effects for the host [18, 93, 118]. Bacteriocins of enterococci are key for their ability to colonize symbiotic communities [159], and direct competence among bacterial strains via antimicrobials has a central role in the configuration of stable microbiomes [160–163]. Therefore, vertically transmitted *Peptoniphilus* and *Helcococcus* may be host-controlled symbionts conditioning [164] the establishment of particular enterococci and/or other symbionts with high competitive ability within the glands of hoopoes, either directly or indirectly. The hoopoe uropygial gland may thus be an organ specialized in providing a space facilitating competence among antimicrobial producers, with safety for the host (outside the digestive tract), to get the best producers of these antimicrobial metabolites for use on body and egg surfaces (Fig. 1c).

The variety of types of organization of uropygial glands in birds is huge, including the location of cavities storing the secretion (lobes or papilla), connection between lobes and papilla, and types of lining of the ducts and cavities (with or without layers of corneous laminae) [65, 143, 154]. Evidence suggesting presence of bacterial communities in the uropygial gland of other bird species is accumulating, and similar host-control mechanisms mediated by gland structure and function, as those suggested for hoopoes, may be widespread in the avian phylogeny.

## Conclusions

In conclusion, the hoopoe uropygial gland is an organ specialized in culturing symbiotic bacteria, at least by compartmentalizing and nurturing a specific microbial assemblage in the papilla, to obtain its special bacteria-processed secretion that the bird uses in several ecological contexts. The patterns described here, supporting that the uropygial gland of European hoopoes is a symbiotic organ, might also appear in other exocrine glands of terrestrial vertebrates known to harbor bacteria (e.g., odor glands of mammals [24, 165–169]). Future studies should consider them as a type of *symbiotic organ* in animals. The uropygial gland of the hoopoe is a key model for the study of symbiotic organs selecting for defensive bacteria.

## Supplementary Information

Below is the link to the electronic supplementary material.

Supplementary Material 1

Supplementary Material 2

Supplementary Material 3

## Data Availability

The datasets generated and/or analysed during the current study are available in the [NCBI] repository, [PRJNA1364298 [ID 1364298 - BioProject - NCBI](https://www.ncbi.nlm.nih.gov/bioproject/1364298)] and [Digibug] repository [(https://hdl.handle.net/10481/108046)].
