# Supplementary material for: The uropygial gland of the European hoopoe as a symbiotic organ
Source: Anim Microbiome. 2026 Apr 30;8:55. doi: 10.1186/s42523-026-00543-y (PMC13130528; doi:10.1186/s42523-026-00543-y)
Supplement: Supplementary file 1 — Supplementary Material 1 [file 42523_2026_543_MOESM1_ESM.docx]

**The uropygial gland of the European hoopoe as a *symbiotic organ***

*Manuel Martín-Vivaldi, Ángela Martínez-García, Juan M. Peralta-Sánchez, Michael Schaub, Raphaël Arlettaz, Antonio M. Martín-Platero, Ester Martínez-Renau,* *María Dolores Barón, Magdalena Ruiz-Rodríguez, Estefanía López-Hernández, Manuel Martínez-Bueno, Eva Valdivia, Juan J. Soler.*

**Additional file 1**

LABORATORY METHODS

**Characterization of bacterial communities**

*ARISA method*

ARISA amplifies an intergenic transcribed spacer (ITS) region between the prokaryotic 16S and 23S rDNA. This region is highly variable both in size and sequence between species, offering higher taxonomic resolution than other fingerprinting techniques [1]. The ITS was amplified using the primer pair ITSF (5´-GTCGTAACAAGGTAGCCGTA-3´) and ITSReub (5´-GCCAAGGCATCCACC-3´) [2]. The primer ITSReub was labeled fluorescently with 6-FAM. Amplifications were performed in 50 µl reaction volumes containing ultrapure H_2_O, 20 µl of 5 PRIME MasterMix (2.5x) including 1.5mM Mg (OAC)_2_, 200 µM dNTPs, 1.25 U Taq DNA polymerase, 0.2 µM of primers and 5 µl of DNA (diluted 1:10). PCRs were conducted in the Eppendorf Mastercycler Nexus Family. Fragments were amplified under the following conditions: initial denaturation at 94° C for 2 min, followed by 30 cycles with denaturation at 94 °C for 45 s, annealing at 52° C for 45 s, and extension at 72 °C for 1 min, with a final extension at 72 °C for 5 min. Amplified PCR products were diluted 1:10 and denatured by heating in formamide. Fragment lengths were determined by automated fluorescent capillary electrophoresis in a 3130 Genetic Analyzer. Electropherogram peak values were calculated after interpolation with an internal size standard named GeneScan™ 1200 LIZ dye Size Standard (Applied Biosystems). These analyses were performed in the Scientific Instrumentation service of Granada University.

Peak Scanner 1.0 (Applied Biosystems) was used to determine fragment length in terms of base pairs of each peak, that enables the identification of different bacterial OTUs (i.e., ITSs) within each sample. Due to measurement variability across runs, we binned DNA fragment lengths by using available scripts in R-environment [http://cran.r-project.org/] at <https://www.mpi-bremen.de/en/Softwares.html> [3] with a window size of 4 base pairs (bp) and a distance of two consecutive binning frames (i.e., shift) of 0.1. All samples from both populations were binned together. All peaks with RFI (Relative Fluorescence Intensity) values of < 0.09% were not included in further analyses since they consisted of background peaks. Only fragments above a threshold of 50 fluorescence units and ranging between 100 and 1,000 bp were taken into consideration to include the maximum number of peaks while excluding background fluorescence [3]. We used the presence-absence matrix generated after the binning process for all analyses.

When all sampled bacterial communities (104 ITSs) were considered, the estimated prevalence of most ARISA peaks proved very low. To reduce the effect of rare bacterial strains on the analysis of similarities between different bacterial communities, we considered only the 63 ITSs that appeared in at least 10% of individuals in one sample type. Length of these ITS fragments ranged between 182 bp and 622 bp (Additional material 3: Fig. S1).

*16S rRNA amplicon sequencing method*

Amplicon libraries were constructed by a two-step PCR approach: first, a PCR was performed from bacterial total DNA of the V6-V8 region of the 16S rRNA gene using the primer pair B969F (5´-ACGCGHNRAACCTTACC-3´) and BA1406R (5´- ACGGGCRGTGWGTRCAA-3´) [4]; and second, a PCR was applied to add barcodes to individual samples, so that the derived sequences can be sorted into respective samples in downstream analysis. These barcodes overlap with the sequence of the primers used in the first PCR. Purification steps were carried out using HigherPurity™ DNA Purification SPRI Magnetic Beads (Canvax®). Then, DNA concentration was measured using a Qubit® Fluorimeter (Invitrogen^TM^) and standardized to 25 ng of DNA per sample in the sequencing mix. All samples from both populations were processed together. High throughput sequencing was carried out in Illumina MiSeq platform at the Integrated Microbiome Resource, Centre for Comparative Genomics and Evolutionary Bioinformatics (CGEB), University of Dalhousie (Canada).

The processing of the sequences obtained from 16S rRNA amplicon sequencing was carried out with QIIME2 v2021.11 [Quantitative Insights in Microbial Ecology, 5]. First, primer trimming was performed using cutadapt plugin [6]. Forward and reverse reads were joined using VSEARCH [7]. Quality filtering was performed using default parameters, and, afterwards, we used Deblur, a sub-operational-taxonomic-unit approach, in order to create the ASV table and remove sequencing errors [8]. Sequences that passed quality filters were truncated to 402 bp, using a quality score of 20 as a threshold. Then, we used the fragment insertion plugin, a script that performs the sequences alignment and creates the bacterial phylogenetic tree [9] using sepp-refs-silva-128. Taxonomic assignment was performed training a classifier on silva-138-99-tax database for the V6-V8 region of 16S rRNA genes [10]. Finally, chloroplasts, mitochondria, non-phylum-assigned and non-bacterial DNA were removed from the ASV table, retaining only bacterial ASVs. For several types of sequence analyses it is recommended to work only with features over a minimum prevalence [11], so we decided to retain those present in at least 3 samples. The available readings per sample were rarefied to obtain an even sampling for diversity estimates and community composition comparisons. To retain as many samples as possible, sampling depth was determined by the lowest number of readings found in eggshells (3864 reads). We only lost one Swiss secretion sample with this depth. In this way, a total of 324,576 reads and 661 ASVs were retained for subsequent analyses.

**Sample preparation for Fluorescence In Situ Hybridization (FISH)**

Gland sections were hybridized several months after collection. To permeate cell walls, 10 μL of lysozyme solution (100 mM Tris hydrochloride (Tris-HCl); 50 mM ethylenediaminetetraacetic acid (EDTA); pH 8; 2 mg/mL lysozyme, USB Corporation, Cleveland, OH, USA) were added to each slide and incubated at 37 °C for 60 min in a humid atmosphere. Then, slides were washed with filtered distilled water, air-dried, and dehydrated in successive 50%, 80%, and 96% ethanol baths. Pre-hybridization was performed by dropping 5 μL of Dig Easy Hib detergent (Roche, Basel, Switzerland) and incubating slides at 45 °C for 2 h in a wet atmosphere. To wash the detergent, slides were immersed in pre-hybridization buffer (20 mM Tris-HCl; 0.9 M NaCl; sodium dodecyl sulfate (SDS) 0.01%; pH 7.2–7.4) and in filtered distilled water, and then dried at 28 °C. The universal probe Eub338 (5’-GCTGCCTCCCGTAGGAGT-3′ [12] labeled with Cy3 (Biomers.net, Ulm, Germany) was used for bacteria detection. Hybridization was performed by adding 30 μL of the probe-containing hybridization buffer (1 μL probe; 20 mM Tris-HCl; 0.9 M NaCl; SDS 0.1%; formamide 25%, Sigma-Aldrich; pH 7.2–7.4) to the slides and incubating at 45 °C overnight in a wet atmosphere. Non-specific hybridization was controlled by increasing the temperature to 48 °C for 20 min in a wet atmosphere. Non-hybridized probes were washed away by immersing the slides successively in hybridization buffer and distilled water for 2 min. Slides were air-dried at room temperature. Hybridization buffers were pre-heated to ensure that SDS was dissolved in high NaCl concentration. Afterwards, buffers were kept at room temperature.

After hybridization, 10 μL Hoechst (10 μg/mL, Sigma-Aldricht, St. Louis, MO, USA) were added to slides and the mixture was maintained for 30 min in a wet atmosphere to stain DNA as positive control for the presence of bacteria and host cells. Then, slides were washed with phosphate-buffered saline (PBS) for 2 min and air-dried at room temperature. Slides were mounted by applying a drop of antifading solution (Vectashield Mounting Medium H-1000, Vector Laboratories, Burlingame, CA, USA) and a coverslip, and then sealed with varnish. The whole process was performed maintaining slides in the dark to avoid fading of the fluorescent probes and Hoechst dye, and they were stored in dark boxes at 4 °C until visualization under fluorescence microscopy.

References

1. Danovaro, R., Luna, G.M., Dell'Anno, A., and Pietrangeli, B. Comparison of two fingerprinting techniques, terminal restriction fragment length polymorphism and automated ribosomal intergenic spacer analysis, for determination of bacterial diversity in aquatic environments*.* *Applied and Environmental Microbiology*, 2006. 72(9): 5982-5989.

2. Cardinale, M., Brusetti, L., Quatrini, P., Borin, S., Puglia, A.M., Rizzi, A., Zanardini, E., Sorlini, C., Corselli, C., and Daffonchio, D. Comparison of different primer sets for use in automated ribosomal intergenic spacer analysis of complex bacterial communities*.* *Applied and Environmental Microbiology*, 2004. 70(10): 6147-6156.

3. Ramette, A. Quantitative community fingerprinting methods for estimating the abundance of operational taxonomic units in natural microbial communities*.* *Applied and environmental microbiology*, 2009. 75(8): 2495-2505.

4. Comeau, A.M., Li, W.K., Tremblay, J.-É., Carmack, E.C., and Lovejoy, C. Arctic Ocean microbial community structure before and after the 2007 record sea ice minimum*.* *PloS one*, 2011. 6(11): e27492.

5. Bolyen, E., Rideout, J.R., Dillon, M.R., Bokulich, N.A., Abnet, C.C., Al-Ghalith, G.A., Alexander, H., Alm, E.J., Arumugam, M., Asnicar, F., Bai, Y., Bisanz, J.E., Bittinger, K., Brejnrod, A., Brislawn, C.J., Brown, C.T., Callahan, B.J., Caraballo-Rodríguez, A.M., Chase, J., Cope, E.K., Da Silva, R., Diener, C., Dorrestein, P.C., Douglas, G.M., Durall, D.M., Duvallet, C., Edwardson, C.F., Ernst, M., Estaki, M., Fouquier, J., Gauglitz, J.M., Gibbons, S.M., Gibson, D.L., Gonzalez, A., Gorlick, K., Guo, J., Hillmann, B., Holmes, S., Holste, H., Huttenhower, C., Huttley, G.A., Janssen, S., Jarmusch, A.K., Jiang, L., Kaehler, B.D., Kang, K.B., Keefe, C.R., Keim, P., Kelley, S.T., Knights, D., Koester, I., Kosciolek, T., Kreps, J., Langille, M.G.I., Lee, J., Ley, R., Liu, Y.-X., Loftfield, E., Lozupone, C., Maher, M., Marotz, C., Martin, B.D., McDonald, D., McIver, L.J., Melnik, A.V., Metcalf, J.L., Morgan, S.C., Morton, J.T., Naimey, A.T., Navas-Molina, J.A., Nothias, L.F., Orchanian, S.B., Pearson, T., Peoples, S.L., Petras, D., Preuss, M.L., Pruesse, E., Rasmussen, L.B., Rivers, A., Robeson, M.S., Rosenthal, P., Segata, N., Shaffer, M., Shiffer, A., Sinha, R., Song, S.J., Spear, J.R., Swafford, A.D., Thompson, L.R., Torres, P.J., Trinh, P., Tripathi, A., Turnbaugh, P.J., Ul-Hasan, S., van der Hooft, J.J.J., Vargas, F., Vázquez-Baeza, Y., Vogtmann, E., von Hippel, M., Walters, W., Wan, Y., Wang, M., Warren, J., Weber, K.C., Williamson, C.H.D., Willis, A.D., Xu, Z.Z., Zaneveld, J.R., Zhang, Y., Zhu, Q., Knight, R. and Caporaso, J.G. Reproducible, interactive, scalable and extensible microbiome data science using QIIME 2*.* *Nature Biotechnology*, 2019. 37(8): 852-857.

6. Martin, M. Cutadapt removes adapter sequences from high-throughput sequencing reads*.* *EMBnet.journal; Vol 17, No 1: Next Generation Sequencing Data AnalysisDO - 10.14806/ej.17.1.200*, 2011.

7. Rognes, T., Flouri, T., Nichols, B., Quince, C., and Mahé, F. VSEARCH: a versatile open source tool for metagenomics*.* *PeerJ*, 2016. 4: e2584.

8. Amir, A., McDonald, D., Navas-Molina, J.A., Kopylova, E., Morton, J.T., Zech Xu, Z., Kightley, E.P., Thompson, L.R., Hyde, E.R., Gonzalez, A., and Knight, R. Deblur rapidly resolves single-nucleotide community sequence patterns. *mSystems*, 2017. 2(2): e00191-00116.

9. Janssen, S., McDonald, D., Gonzalez, A., Navas-Molina, J.A., Jiang, L., Xu, Z.Z., Winker, K., Kado, D.M., Orwoll, E., Manary, M., Mirarab, S., and Knight, R. Phylogenetic placement of exact amplicon sequences improves associations with clinical information. *mSystems*, 2018. 3(3): e00021-00018.

10. Werner, J.J., Koren, O., Hugenholtz, P., DeSantis, T.Z., Walters, W.A., Caporaso, J.G., Angenent, L.T., Knight, R., and Ley, R.E. Impact of training sets on classification of high-throughput bacterial 16s rRNA gene surveys*.* *Isme j*, 2012. 6(1): 94-103.

11. Morton, J.T., Sanders, J., Quinn, R.A., McDonald, D., Gonzalez, A., Vázquez-Baeza, Y., Navas-Molina, J.A., Song, S.J., Metcalf, J.L., Hyde, E.R., Lladser, M., Dorrestein, P.C., and Knight, R. Balance trees reveal microbial niche differentiation. *mSystems*, 2017. 2(1): e00162-00116.

12. Amann, R.I., Binder, B.J., Olson, R.J., Chisholm, S.W., Devereux, R., and Stahl, D.A. Combination of 16S rRNA-targeted oligonucleotide probes with flow cytometry for analyzing mixed microbial populations*.* *Applied and Environmental Microbiology*, 1990. 56(6): 1919-1925.
