# Supplementary material for: The uropygial gland of the European hoopoe as a symbiotic organ
Source: Anim Microbiome. 2026 Apr 30;8:55. doi: 10.1186/s42523-026-00543-y (PMC13130528; doi:10.1186/s42523-026-00543-y)
Supplement: Supplementary file 2 — Supplementary Material 2 [file 42523_2026_543_MOESM2_ESM.docx]

**The uropygial gland of the European hoopoe as a *symbiotic organ***

*Manuel Martín-Vivaldi, Ángela Martínez-García, Juan M. Peralta-Sánchez, Michael Schaub, Raphaël Arlettaz, Antonio M. Martín-Platero, Ester Martínez-Renau,* *María Dolores Barón, Magdalena Ruiz-Rodríguez, Estefanía López-Hernández, Manuel Martínez-Bueno, Eva Valdivia, Juan J. Soler.*

**Additional file 2**

TABLES

| **Table S1.** Results of Kruskal-Wallis analyses comparing, among sample types, the relative abundances of the main bacterial phyla present in European hoopoe females’ uropygial secretions, nestlings’ uropygial secretions and eggshells. The post-hoc tests for the comparison between nestlings’ and females’ secretions are included. | | | | | |
| --- | --- | --- | --- | --- | --- |
|  | | *Kruskal-Wallis test* | | Nestlings’ *vs* females’ secretions  (post-hoc) |  |
| *Phyla* |  | *H(2,84)* | *p* | *p* |  |
| Ph, *Firmicutes* | | 11.97 | **0.003** | 0.194 |  |
| Ph*. Bacteroidota* | | 4.80 | 0.091 | 0.185 |  |
| Ph*. Actinobacteria* | | 23.24 | **0.00001** | 0.554 |  |
| Ph. *Proteobacteria* | | 49.10 | **0.00001** | 0.993 |  |
| Ph*. Campylobacterota* | | 1.06 | 0.588 | 0.849 |  |

| **Table S2.** Results of GLM analyses comparing three alpha diversity estimates, using 16S rRNA amplicon sequencing ASVs, among the three types of samples (eggshells, nestlings’ uropygial secretions and females’ uropygial secretions), in the Spanish and Swiss European hoopoe populations. The whole model as well as the pair-wise tests comparing the same sample type between populations (factor 1x2 within sample type) are presented. | | | | | | | |
| --- | --- | --- | --- | --- | --- | --- | --- |
| *GLM* | | | | | | |  |
| *Whole model for* ***nº observed ASVs*** | | |  |  |  |  |  |
| *Factor* |  | | *Nested in* | *df* | *F* | *p* |  |
| 1) Population | Fixed | |  | 1 | 30.5 | **0.00001** |  |
| 2) Sample type | Fixed | |  | 2 | 299.1 | **0.00001** |  |
| 3) Nest | Random | | Country | 22 | 1.5 | 0.136 |  |
| 1x2 | Fixed | |  | 2 | 25.8 | **0.00001** |  |
| 2x3 | Random | |  | 33 | 20.6 | **0.00001** |  |
| Res. |  | |  | 21 |  |  |  |
| *Pair-Wise tests (1x2)* | | |  |  |  |  |  |
| *Factor* | *Between* | | |  |  | *Tukey test*  *p* |  |
| Population | Eggshells | | |  |  | **0.0015** |  |
| Population | Nestlings’ secretions | | |  |  | 0.983 |  |
| Population | Females’ secretions | | |  |  | 0.143 |  |
| *GLM* | | | | | | | |
| *Whole model for* ***faith phylogenetic diversity*** | | | |  |  |  |  |
| *Factor* | |  | | *Nested in* | *df* | *F* | *p* |
| 1) Population | | Fixed | |  | 1 | 17.7 | **0.0003** |
| 2) Sample type | | Fixed | |  | 2 | 353.9 | **0.00001** |
| 3) Nest | | Random | | Country | 22 | 1.6 | 0.108 |
| 1x2 | | Fixed | |  | 2 | 13.3 | **0.00006** |
| 2x3 | | Random | |  | 33 | 4.0 | **0.00072** |
| Res. | |  | |  | 21 |  |  |
| *Pair-Wise tests (1x2)* | | | |  |  |  |  |
| *Factor* | | *Between* | | |  |  | *Tukey test*  *p* |
| Population | | Eggshells | | |  |  | **0.0015** |
| Population | | Nestlings’ secretions | | |  |  | 0.710 |
| Population | | Females’ secretions | | |  |  | 0.708 |
| *GLM* | | | | | | | |
| *Whole model for* ***Berger-Parker dominance*** | | | |  |  |  |  |
| *Factor* | |  | | *Nested in* | *df* | *F* | *p* |
| 1) Population | | Fixed | |  | 1 | 1.9 | 0.183 |
| 2) Sample type | | Fixed | |  | 2 | 6.5 | **0.014** |
| 3) Nest | | Random | | Country | 22 | 0.9 | 0.595 |
| Res. | |  | |  | 56 |  |  |
| *Pair-Wise tests* ***sample type*** | | | |  |  |  |  |
| *Between* | |  | | |  |  | *Tukey test*  *p* |
| Nestlings’ secretions | | | Eggshells | |  |  | **0.0015** |
| Nestlings’ secretions | | | Females’ secretions | |  |  | 0.110 |
| Females’ secretions | | | Eggshells | |  |  | 0.438 |

| **Table S3.** Results of a PERMANOVA analysis comparing the composition of bacterial communities of the three sample types (eggshells, nestlings’ uropygial secretions and females’ uropygial secretions), in the Spanish and Swiss populations. The matrix of distances among samples used is based on Jaccard similarity calculated with presence/absence of the 63 most prevalent ARISA ITSs. The whole model as well as the pair-wise tests for the interaction population x sample type are presented. For pair-wise comparisons, differences in beta dispersions using a Permdisp analysis are presented in last column. | | | | | | | | |
| --- | --- | --- | --- | --- | --- | --- | --- | --- |
| ***Permanova*** | | | | | | | | |
| ***Whole model*** | | | | | | | |  |
| ***Factor*** |  | ***Nested in*** | | ***df*** | | ***PseudoF*** | ***p*** |  |
| 1) Population | Fixed |  | | 1 | | 6.90 | **0.0001** |  |
| 2) Sample type | Fixed |  | | 2 | | 10.53 | **0.0001** |  |
| 3) Nest | Random | Population | | 43 | | 3.63 | **0.0001** |  |
| 1x2 | Fixed |  | | 2 | | 4.08 | **0.0001** |  |
| 2x3 | Random |  | | 83 | | 1.80 | **0.0001** |  |
| Res. |  |  | | 120 | |  |  |  |
|  |  | |  | |  |  |  |  |
| ***Pair-Wise tests (1x2)*** | | |  | |  |  |  | ***Permdisp*** |
| ***Factor*** | ***Within*** | | | | ***Similarity*** | ***t*** | ***p*** | ***p*** |
| Population | Eggshells | | | | 11.89 | 1.48 | **0.007** | 0.513 |
| Population | Nestlings’ secretions | | | | 21.06 | 2.89 | **0.0001** | **0.034** |
| Population | Females’ secretions | | | | 19.07 | 2.94 | **0.0001** | 0.648 |
| ***Factor*** | ***Within Spain*** | | | | ***Similarity*** | ***t*** | ***p*** | ***p*** |
| Sample type | Eggshells-Fem. Secret. | | | | 6.21 | 3.46 | **0.0001** | **0.001** |
| Sample type | Eggshells-Nestl. Secret. | | | | 7.82 | 2.80 | **0.001** | **0.001** |
| Sample type | Fem.-Nestl. Secretions | | | | 27.96 | 1.66 | **0.01** | 0.343 |
| ***Factor*** | ***Within Switzerland*** | | | | ***Similarity*** | ***t*** | ***p*** | ***p*** |
| Sample type | Eggshells-Fem. Secret. | | | | 13.11 | 3.07 | **0.0001** | **0.001** |
| Sample type | Eggshells-Nestl. Secret. | | | | 13.30 | 2.99 | **0.0001** | **0.001** |
| Sample type | Fem.-Nestl. Secretions | | | | 27.09 | 1.23 | 0.142 | 0.867 |

| **Table S4.** Relationship between the prevalences of bacterial 16S rRNA amplicon sequencing ASVs in Spain and Switzerland, for three types of European hoopoe samples (eggshells, nestlings’ uropygial secretions and females’ uropygial secretions). Analyses are GLZ models with Poisson distribution, with prevalence in Switzerland as dependent variable. The whole model as well as one considering only uropygial secretions are presented. | | | | | | | | | | |  |
| --- | --- | --- | --- | --- | --- | --- | --- | --- | --- | --- | --- |
|  |  | *Whole model* | | |  |  | |  | |  |  |
| *Factor* |  |  | | | *df* | *Wald* | | *p* | |  |  |
| 1) Prevalence Spain | Covariate |  | | | 1 | 7562.19 | | **0.0001** | |  |  |
| 2) Sample type | Fixed |  | | | 2 | 2835.01 | | **0.0001** | |  |  |
| 1x2 | Fixed |  | | | 2 | 1757.14 | | **0.0001** | |  |  |
|  |  |  | | |  |  | |  | |  |  |
|  |  | *Only secretions* | | |  |  | |  | |  |  |
| *Factor* |  |  | | | *df* | *Wald* | | *p* | |  |  |
| 1) Prevalence Spain | Covariate |  | | | 1 | 6830.00 | | **0.000001** | |  |  |
| 2) Sample type | Fixed |  | | | 1 | 0.03 | | 0.855 | |  |  |
| 1x2 | Fixed |  | | | 1 | 1.33 | | 0.250 | |  |  |
|  |  |  | | |  |  | |  | |  |  |
|  |  | |  |  | | |  | |  | | |
|  |  | |  |  | | |  | |  | | |

**Figure S1.** Differences in the number of ARISA ITSs among the three sample types for both populations. The interaction between sample type and population was significant in a GLM model with similar design to that in Additional file 2 Table 2 (1x2, F_2,113.9_ = 23.5, p < 0.0001, whole model not shown).

**
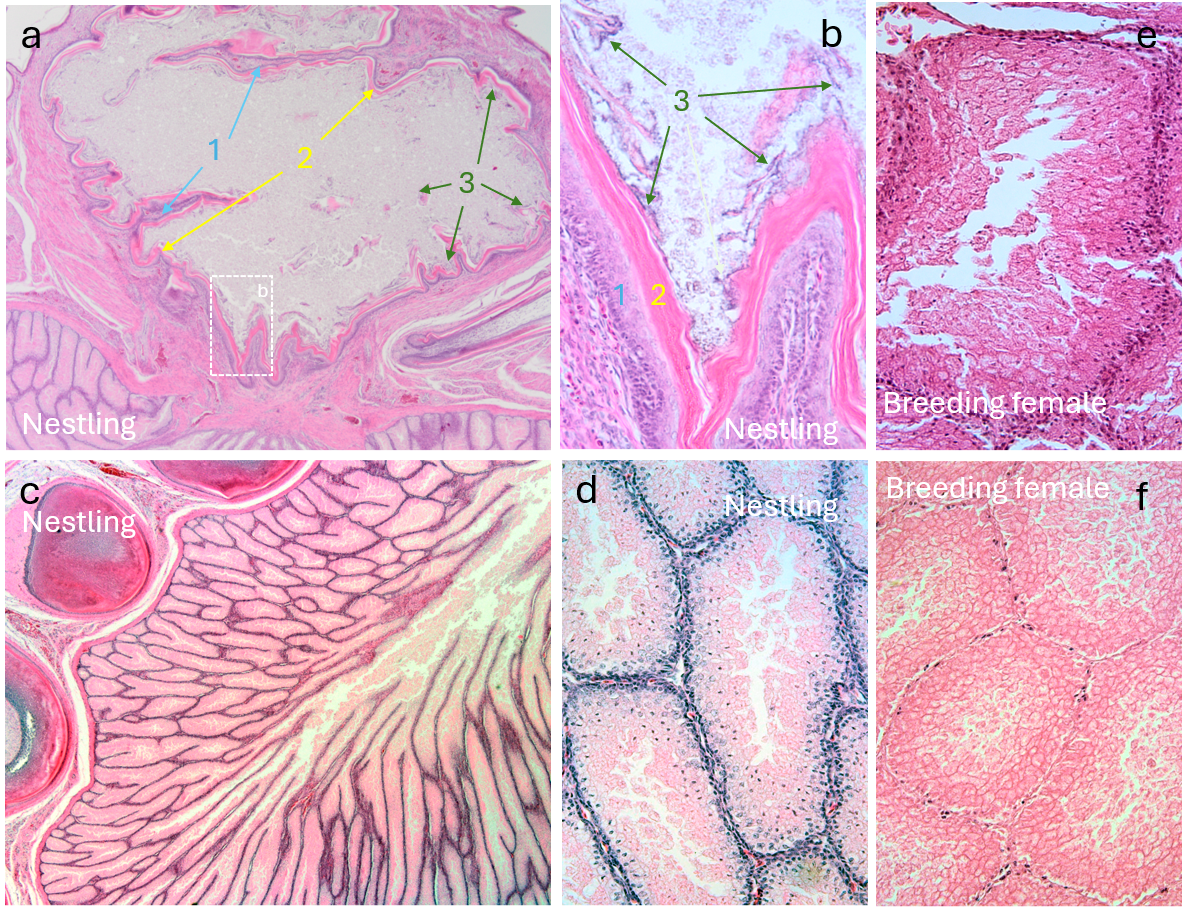
**

**Figure S2.** Anatomy of the uropygial gland of a hoopoe nestling (**a-c**) and detailed appearance of the cells in the secretory tubules of the same nestling (**d**), and two breeding females (**e-f**, **f** corresponds to the gland of the same female included in Fig. 4 in the main text). All are individuals in the phase producing dark secretions (see Fig. 7-b in the main text). Image **b** shows details of the internal wall of the papilla at the site indicated by the dashed line in image **a**. Image **c** shows the connection of secretory tubules with the main collector cavity in one of the secretory lobes of the gland, which is filled with secreted material. Numbers mark: **1** basal and proliferative layers of the epithelium of the ampulla wall, **2** corneous layer of the ampulla wall, **3** masses of bacteria (thin grains violet-stained). In images **d-f** there are no oil droplets inside cells in the intermedium-transitium layers of the glandular epithelium (compare with images c and f in Additional file 2: Fig. S3). All images are hematoxylin-eosin-stained semi fine sections of glands. Images a and c 40x, images b, d, e, f 400x.

**
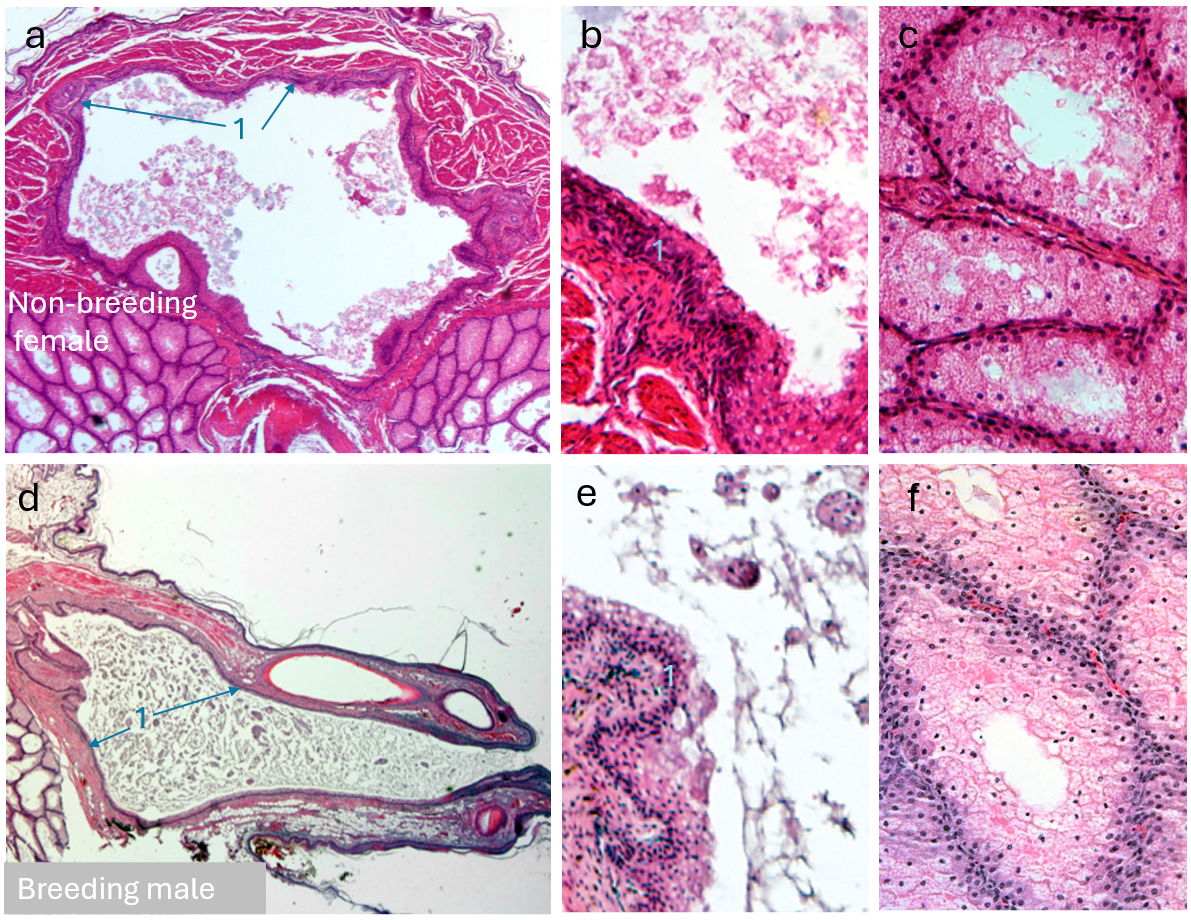
**

**Figure S3.** Anatomy of the uropygial gland of hoopoe individuals in phases producing white secretion: (**a-b**) a non-breeding hoopoe female and (**d-e)** a breeding male, both of which maintained some material inside the papilla after the fixing process (both are different individuals from those included in Fig. 4 in the main text). Images **b** and **e** show details of the internal wall of the papilla of the same individuals. In **c** and **f** it is shown the detailed appearance of the cells in the secretory tubules of hoopoes producing white secretions (see Fig. 4-i in the main text): **c** corresponds to the same female gland of image **a**, **f** to the same male of Fig. 4-h in the main text. Numbers mark: **1** basal and proliferative layers of the epithelium of the ampulla wall. There is not any apparent keratinized corneous layer in the epithelium of the internal ampulla wall. In images **c** and **d** it is very apparent the abundance of oil droplets inside cells in the intermedium-transitium layers of the glandular epithelium. All images are pictures of hematoxylin-eosin-stained sections of glands. Images a, d 40x, images b, c, e, f 400x.
