# Supplementary material for: The uropygial gland of the European hoopoe as a symbiotic organ
Source: Anim Microbiome. 2026 Apr 30;8:55. doi: 10.1186/s42523-026-00543-y (PMC13130528; doi:10.1186/s42523-026-00543-y)
Supplement: Supplementary file 3 — Supplementary Material 3 [file 42523_2026_543_MOESM3_ESM.docx]

**The uropygial gland of the European hoopoe as a *symbiotic organ***

*Manuel Martín-Vivaldi, Ángela Martínez-García, Juan M. Peralta-Sánchez, Michael Schaub, Raphaël Arlettaz, Antonio M. Martín-Platero, Ester Martínez-Renau,* *María Dolores Barón, Magdalena Ruiz-Rodríguez, Estefanía López-Hernández, Manuel Martínez-Bueno, Eva Valdivia, Juan J. Soler.*

**Additional file 3**

*Relationship between ITSs and ASVs*

Due to rRNA gene copy number variation among microbial taxa, several ITS peaks can be measured on a particular bacterial strain, and distantly related bacterial taxa can occasionally have ITSs of similar length despite their difference in sequence [1-3]. Nevertheless, in a particular community, the ITSs may serve as markers of particular taxa. For example, *Enterococcus faecalis* isolated from the hoopoe uropygial glands, particularly strain MRR10 [4] typically produces two ITS peaks, around lengths 306 and 406, when amplified with primers ItsF and ItsReub (see below). To better understand our current results and previous research with ITSs on hoopoe uropygial secretion bacterial symbionts, we explored the correspondence between the most important ITS sizes and the ASVs for the subset of samples that were studied with both methods in our Spain-Switzerland comparison. Given the high prevalence of many of the constituents of the core microbiome, instead of using prevalence of ITSs, we have looked for relationships between the relative abundance of the different ITSs within samples (estimated by the proportion of the total area corresponding to each ITS peak) and the relative abundance of ASVs taxonomically identified by 16S rRNA amplicon sequencing. Given that we are comparing each sample with itself, and relative abundances of markers of each taxa should be correlated within each sample, compositional issues do not affect these analyses. For this approach, we used relative abundances of 16S rRNA amplicon sequencing ASVs calculated at genus level. To avoid a disproportionate influence of very abundant genera in the quantitative analyses, the value for each genus in each sample was standardized to the proportion over the total of the genus across the samples analyzed (i.e., total relative abundance of each genus summed 1.00 across samples).

For this approach, the relative abundance of each particular ITS size to be identified was used as the dependent variable and the relative abundance of genera of the 59 constituents of the core microbiome of secretions were used as predictors in Forward Stepwise General Linear Regressions. Our aim was to identify the most prevalent ITS OTUs, as well as those previously found to be key actors in our previous studies on the mode of acquisition of the symbiotic bacteria in hoopoe glands [5, 6] and their transmission among body parts when preening [7]. However, it was apparent that many of these OTUs with high prevalence were surrounded by other ITSs of very similar size (4bp difference) that also reached high frequency (Fig. S1).

**Figure S1.** Comparison of the frequency of appearance of the 63 most prevalent ARISA OTUs per sample type (a: secretions; b: eggshells) and country. OTUs are named by their size in base pairs (bp).

Frequently, the presence and abundance of consecutive ITS sizes were negatively related. For example, the two most prevalent OTUs for all sample types (OT534 and OT566) appeared as markers of groups of samples in opposition to OT538 and OT570, respectively, in all or some of the types of samples. This suggests that pairs 534-538 and 566-570 represent alternative components of the community. In fact, the presence of OT534 in samples was much lower when OT538 was also present than when it was absent, and the same pattern was observed between OT566 and OT570 (Table S1, Fig. S2 a-b).

| **Table S1.** Co-occurrence of OTUs of the pairs (a) OT534-OT538 and (b) OT566-OT570 in samples of hoopoe eggshell and uropygial secretion bacterial communities. The frequency of co-occurrence (2x2 Tables and Chi-square tests) and the influence of country of origin in such co-occurrence (GLZ tests) are analysed. The presence of the complementary OTUs were negatively related in both cases, and only for (b) did this trend differ among countries (significant interaction). | | | | | | |
| --- | --- | --- | --- | --- | --- | --- |
| 1. *Pair OT534-OT538* | | | | | | |
| *2x2 Table* | |  | *GLZ (OT534 dependent)* | | | |
| *OT538* | *OT534 present*  *% (n)* |  | *Factor* | *Estimate* | *Wald* | *P* |
| Present | 51.0 (57) |  | Country (Spain) | -0.530 | 10.59 | 0.001 |
| Absent | 84.1 (251) |  | OT538 (absence) | 0.746 | 20.99 | <0.00001 |
| *Chi-square* | 29.7 |  | Country x OT538 | -0.209 | 1.65 | 0.199 |
| *p* | <0.0001 |  |  |  |  |  |
|  | | | | | | |
| 1. *Pair OT566-OT570* | | | | | | |
| *2x2 Table* | |  | *GLZ (OT566 dependent)* | | | |
| *OT570* | *OT566 present*  *% (n)* |  | *Factor* | *Estimate* | *Wald* | *P* |
| Present | 35.0 (100) |  | Country (Spain) | -0.192 | 1.84 | 0.175 |
| Absent | 80.3 (208) |  | OT570 (absence) | 0.865 | 37.38 | <0.000001 |
| *Chi-square* | 61.4 |  | Country x OT570 | -0.533 | 14.21 | 0.0001 |
| *p* | <0.0001 |  |  |  |  |  |

|    1. ITSs 566-570 | 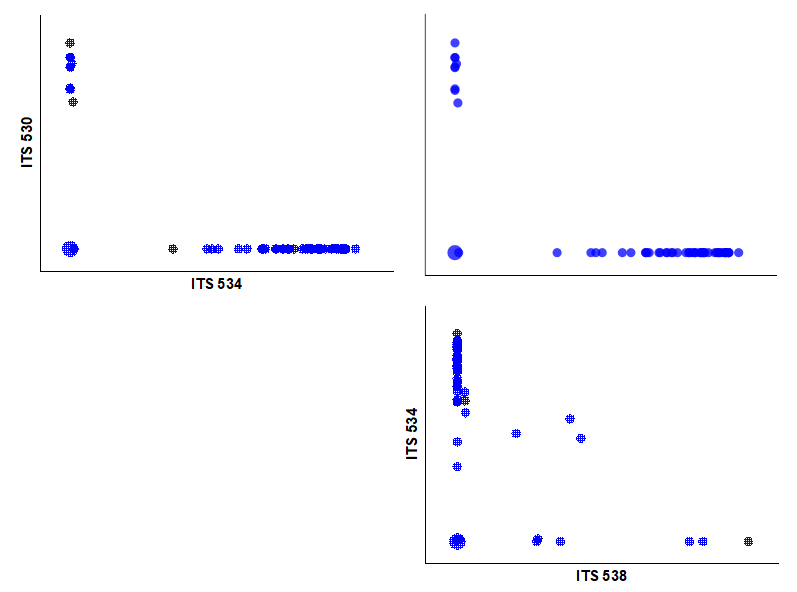   1. ITSs 530 to 538 |
| --- | --- |
| 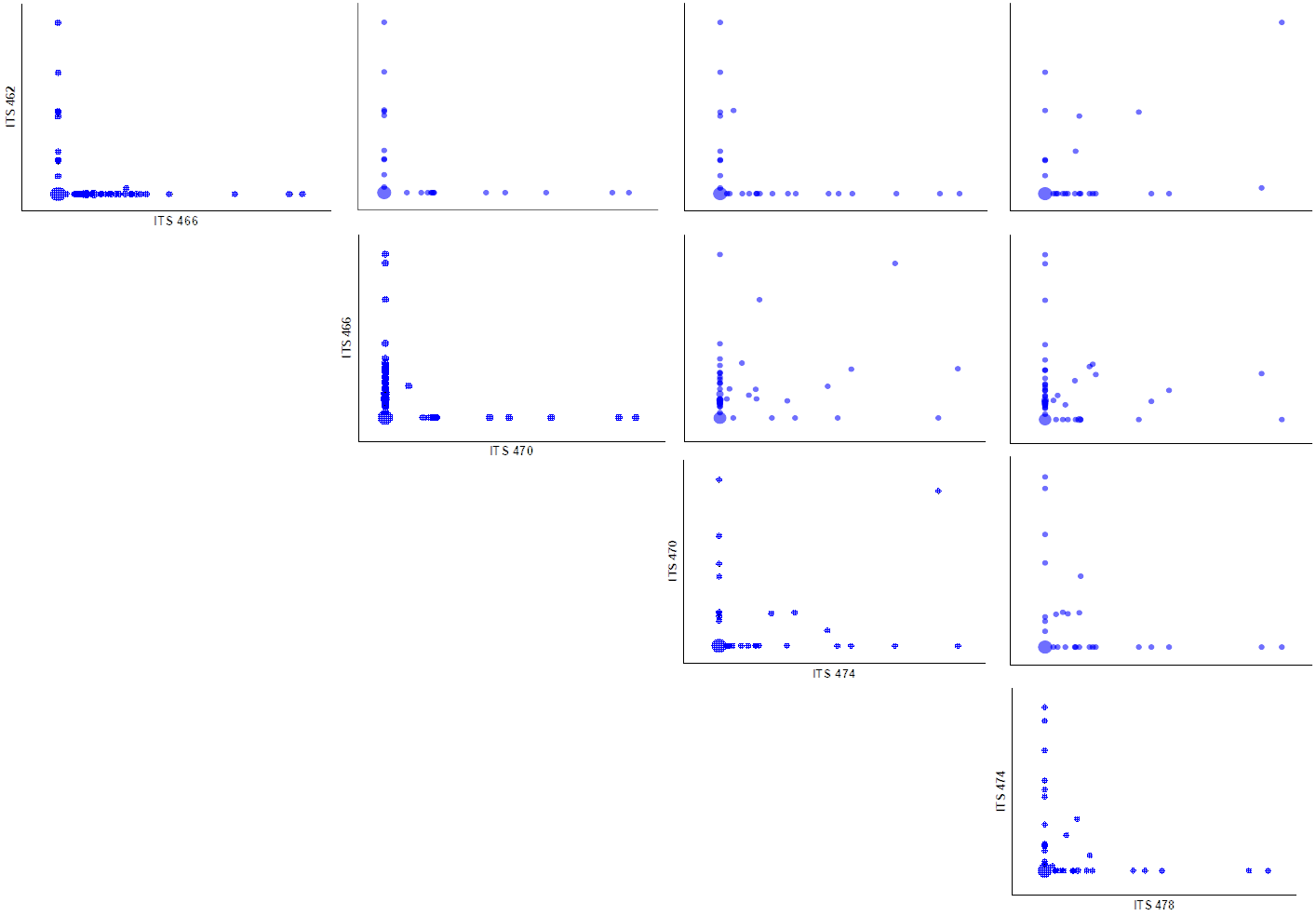   1. ITSs 462 to 478 | |

**Figure S2.** Relationships between the relative abundances of consecutive ITS sizes in the bacterial communities of hoopoe uropygial secretions, examples of (a) two, (b) three, and (c) five consecutive sizes.

An exploration of the correspondence between such consecutive ITS sizes and particular 16S rRNA amplicon sequencing ASVs or genera, suggests that several variants (ASVs) of the same genus may complement each other in explaining a particular ITS size, but also that several consecutive sizes should be added up for better explaining a genus or group of ASVs. Given the large number of ASVs and the difficulty of testing all possible combinations, we performed a first approach analysing relationships between ASVs grouped at genus level and groups of consecutive ITS sizes with added relative abundances. This approach showed that two distantly related genera, both highly ranked in the core microbiome of hoopoe uropygial secretions, *Helcococcus* (Phylum Firmicutes) and *Varibaculum* (Phylum Actinobacteria), together explain the relative abundance of the ITSs in the 462-478 range. Despite analysed one by one *Helcococcus* mainly relate to ITS466 and *Varibaculum* to ITS474, the best fit was obtained when both genera were considered together to explain the whole range (Fig. S3). No other genus or strain was found to explain the consecutive surrounding sizes (data not shown).


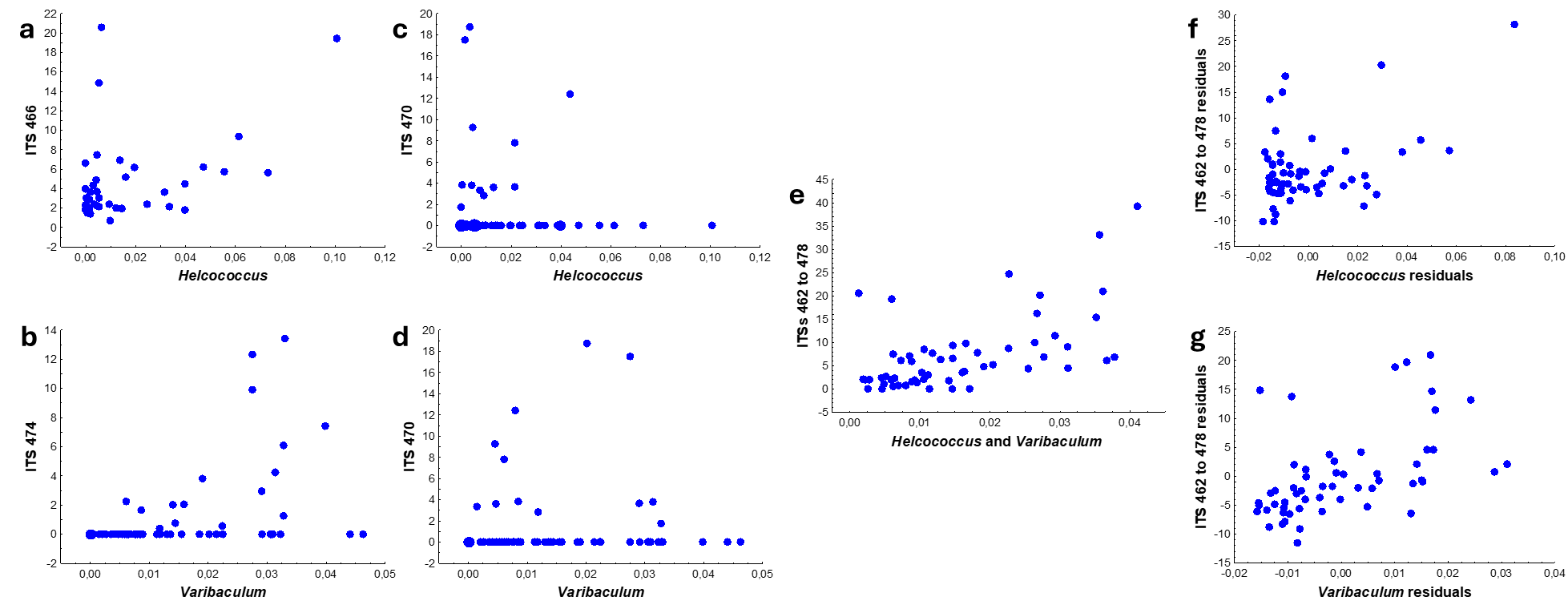


**Figure S3.** Example of a range of consecutive ITS sizes (462 to 478) and their relationships with their main predictors among 16S rRNA amplicon sequencing features at genus level. **a**) Relationship between the relative abundance of ITS 466 and its main predictor as shown by forward stepwise GRM. All samples with *Helcococcus* present also ITS 466 and their relative abundances are positively correlated, however, high abundances of ITS 466 in some samples are not explained by *Helcococcus* abundance. **b**) Relationship between ITS 474 and its main predictor as shown by forward stepwise GRM. All samples with ITS 474 present also *Varibaculum* and their relative abundances are positively correlated, however, many samples show high abundances of *Varibaculum* but do not present ITS 474. **c and d**) ITS 470, intermediate in size between 466 and 474 has not any significant predictor among bacterial genera abundances obtained by 16S rRNA amplicon sequencing, however, there are trends for some samples to show increased abundances of this ITS associated with increased abundances of either *Helcococcu*s (c) or *Varibaculum* (d). **e**) The relative abundance of the 16S rRNA amplicon sequencing features of genera *Helcococcus* and *Varibaculum* considered together was a good predictor of the relative abundance of the group of ITSs between sizes 462 and 478 considered together. **f and g**) Partial contributions of the relative abundances of *Helcococcus* (f) and *Varibaculum* (g) to the relative abundance of the group of ITS between sizes 462 and 478.

To validate this correlative approach, we tested whether the correspondence between peaks 306 and 406 and the genus *Enterococcus* (sizes obtained for *Enterococcus faecalis* by sequencing RISA bands of strain MRR10 and other isolates from hoopoe uropygial glands, Table S2) was detected across our samples. Indeed, we found a significant positive relationship between relative abundance of 16S rRNA amplicon sequencing ASVs identified as enterococci and that of ITSs 306 and 406 (Forward Stepwise GRM, Fig. S4; Table S2). Given the right identification of these peaks, we employed the same method to find the most probable bacterial genera corresponding to the most interesting ITSs (Table S2), i.e., those with high prevalence, those serving as markers of sample types, or those with known interest regarding mode of transmission from previous studies in hoopoes [5-8].

| 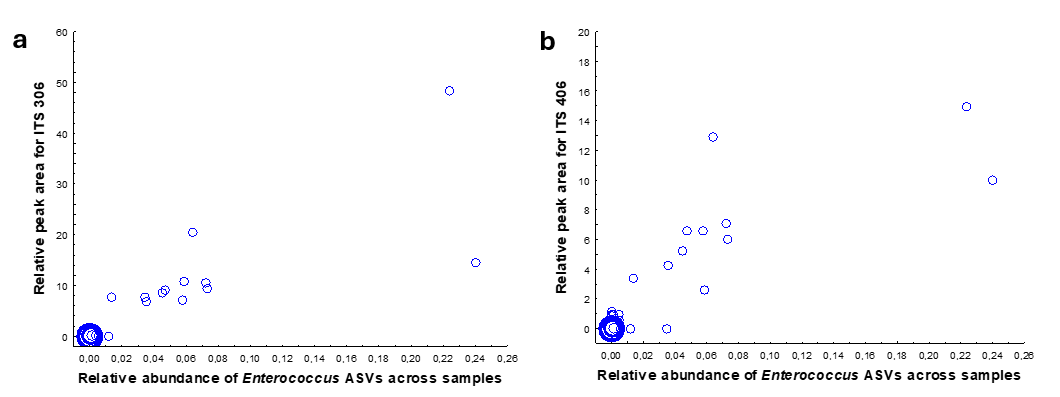 |  |
| --- | --- |
| 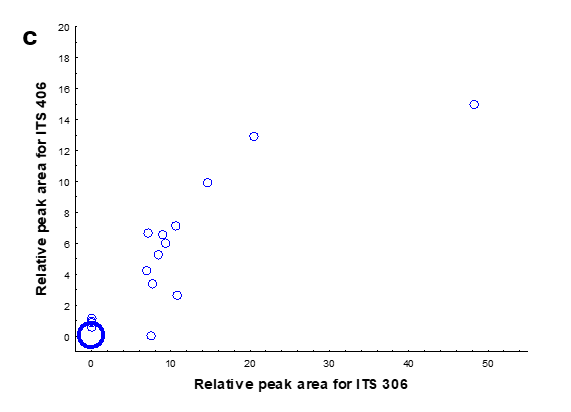 | **Figure S4**. Relationship between the relative peak area of known ITS markers for enterococci (**a**, ITS 306; **b**, ITS 406) and the relative abundance across hoopoe uropygial secretion samples of the sequences assigned to ASVs identified as *Enterococcus sp.* in 16S rRNA amplicon sequencing analyses. In **c** the relationship between both ITSs is depicted. Point sizes reflect number of samples in a log scale. |

As an additional information-source to relate ITS sizes to particular bacterial taxa, we used the sequences previously obtained from RISA bands or clones of the ITS region from hoopoe uropygial secretions [9, 10]. For this approach, the sequences amplified in the original studies with the Primers FGPS1490-72 [11] and FGLP132-38 [12] were used to infer the expected lengths in our ARISA analysis with primers ItsF and ItsReub [13]. To this end, we located each primer pair on the rRNA gene using *Escherichia coli* strain K-12 substr. MG1655 and *Peptoniphilus harei* strain FDAARGOS_1136 as references (Fig. S5), and calculated the corresponding expected lengths. Previous information with a variety of bacteria groups has provided an estimate of differences in ITS sizes for both pairs of primers slightly different from that obtained by us (ARISA peaks 2-5 bp shorter, Martín-Platero, unpublished data). We have considered this possible variation to assign the most probable correspondence of sequenced RISA bands and ITS clones to ARISA peaks.


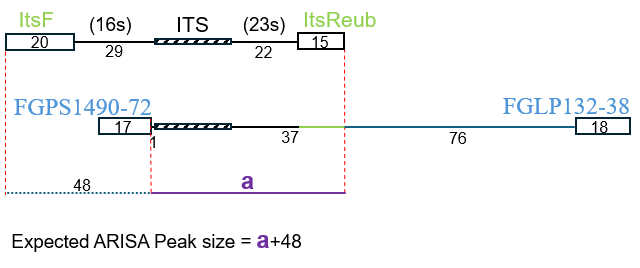


**Figure S5**. Estimation of the expected ARISA Peak sizes with primers ItsF+ItsReub for taxa identified in Rodríguez-Ruano et al [9] from sequences obtained with primers FGLP132-38+FGPS1490-72. The reverse complementary sequence of ItsReub was located within each sequence (green line) to measure size of **a,** and the expected length was calculated by adding the known length of the section between the beginning of ItsF and the end of FGPS1490-72.

The taxonomic assignment of sequences from Rodríguez-Ruano et al (2015) was revised on 21^st^ June 2025 with updated available databases by searching for the closest phylogenetic neighbors using local alignments of our sequences with those in the GenBank Nucleotide collection (nr/nt) database. The MegaBLAST module of the BLAST (Basic Local Alignment Search Tool) Sequence Analysis Tool BLASTN 2.2.30+ (available online at the National Center for Biotechnology Information (NCBI) website (Bethesda, USA, http://www.ncbi.nlm.nih.gov/)) was used. From previous analysis [9] it was evident that the ITS region obtained from hoopoe uropygial gland bacteria usually only presented high homologies with the subregions associated to the tRNA genes for alanine and isoleucine [reviewed in 3]. This means that, in many cases, other regions within the ITSs are not represented within the available databases for strains closely related to those found in hoopoe glands. Indeed, many identifications were based on ITS sections (corresponding to the coding tRNA regions) covering only 16-24% of the query sequence. Since these tRNA genes are usually the most conserved regions [3], differences among candidates in these sequences are small, making it difficult to clearly distinguish closely related genera. To explore which are the most probable homologue regions of our ITSs within the available genomes in the NCBI database, we searched for homologies with the whole sequences and considered candidates not only those strains showing high identities, but also the hits with the highest coverage (Table S2). Additionally, we searched for the same strains with a less stringent criterion for identity (BLASTn) to estimate the probable coverage of the sequences in cases where our strain sequences are distantly related to candidates in the database.

We were able to tentatively identify several of the most interesting ITSs from hoopoe uropygial secretions, given the results of previous studies that used the ARISA approach (Table S2). This will help in interpreting the functioning of this symbiotic organ (see Discussion in main text). Our analysis also suggested that the ITS peaks obtained with ARISA, despite being useful as Operational Taxonomic Units, may hide a complex relationship with actual bacterial taxa. Although correlative, our approach using 16S rRNA amplicon sequencing ASVs suggested that binning may be separating close strains and therefore several close peaks may be variants of the same taxa. On the other hand, in several cases, more than one genus is needed to explain the abundance of a particular peak. Even when using sequencing of ITS clones or isolated fragments from RISA gels, identification based on available sequences in databases usually found only homologies with a short region (usually the most conserved), making that the inference of the most probable taxa is based on small differences in similarity with very low coverage. This causes that, with few exceptions (see sizes 306 and 406 for *Enterococcus*), several candidates, here mainly several *Oscillospiraceae* (synonym of *Hungateiclostridiaceae* in Main Text), *Lachnospiraceae* and *Peptoniphilaceae*, are suitable candidates for the same or very proximal peak sizes, either for their similarity with the sequence, or for their higher coverage (Table S2, last column). This lower sensibility of the ARISA method for distinguishing real taxa, could explain that in some of the analyses comparing populations (see Main text), ARISA did not detect effects so well as 16S rRNA amplicon sequencing.

The frequently low coverage of these ITS sequences when compared with available genomes in NCBI databases, suggests that most bacteria in the hoopoe uropygial gland are not very closely related to the most studied variants, suggesting another level of specialization for this bacterial community (see Discussion in Main Text).

| **Table S2.** List of the main ITS sizes whose most probable taxonomic identification was possible to deduce from correlative analyses relating the ITS relative abundance with the relative abundances of identified 16S rRNA amplicon sequencing ASVs across uropygial secretion samples. For most ITS sizes, the abundance at genus level from 16S rRNA amplicon sequencing was used. For ITSs in the ranges 530-538 and 566-570, both with a very high abundance and prevalence, the analyses were performed with all the particular ASVs of genera with high relative abundance, to get better precision in detecting the influence of predictors. In those cases, the ASVs numbered in bold were those showing significant relationships. Last column shows the most probable identification of ITSs for the estimated sizes of RISA gel-bands or ITS clones based on sequencing performed in Rodríguez-Ruano et al (2015) for the whole uropygial secretion community and Mariscal-Gijón [10] for enterococci. Taxonomic assignments coinciding for correlations and sequencing are highlighted in bold. For the identifications by sequencing bands or clones (last column), high query covers of candidate taxa are highlighted in bold. | | | | | |
| --- | --- | --- | --- | --- | --- |
| ***ITS size*** | ***Interest*** | ***Most probable genus by correlation with 16S rRNA amplicon sequencing ASVs*** | ***Genus ranking in secretions core microbiome (one value per ASV)*** |  | ***Most probable genus by clon/band sequencing***  ***(% query cover, % identity)*** |
| 194 | Prevalence | ***Anaerococcus*** | 6 |  | Unkn. *Paenibacillaceae (67, 80)*  *Peptoniphillaceae*:  *Miniphocaeibacter (58, 73)*  ***Anaerococcus*** *(57, 73)* |
|  |  |  |  |  |  |
| 250 | Prevalence |  | 1, 3, 34, 58 |  | *Peptoniphilus (24, 98)* |
|  |  |  |  |  |  |
|  |  |  |  |  |  |
| 274 | Prevalence | *Anaerococcus* | 6 |  |  |
|  |  |  |  |  |  |
| 278 | Prevalence | *Negativicoccus* | 11 |  |  |
|  |  |  |  |  |  |
|  |  |  |  |  |  |
| 306 | Beneficial, gut origin | ***Enterococcus*** | 28 |  | ***Enterococcus*** *(****100****, 100)* |
|  |  |  |  |  |  |
|  |  |  |  |  |  |
| 338 | Prevalence |  |  |  | Eubacteriales Incertae sedis:  *Levyella (38, 86)*  *Peptoniphillaceae*:  *Murdochiella (38, 84)* ***(76)****  *Lachnospiraceae*:  *Herbinyx* *(22, 90)*  *Coprococcus (21, 90)*  *Faecalimonas (21,90)* |
| 346 | Prevalence,  **female gland origin** |  | 1, 3, 34, 58 |  | Unkn. Bacteria *(****77****, 81)***  *Peptoniphillaceae*:  *Peptoniphilus (53, 98)* ***(78)**** |
|  |  |  |  |  |  |
| 350 | Prevalence |  |  |  | *Actinomycetes*:  *Lawsonella (****94****, 89)*  *Tomitella (31, 92)*  *Dietzia (29, 91)* |
|  |  |  |  |  |  |
| 358 | Prevalence on eggshells |  | 28 |  | *Enterococcus (****100****, 100)* |
|  |  |  |  |  |  |
| 394 | Higher prevalence in Spain |  |  |  | *Veillonellaceae*:  *Megasphaera* *(25, 90)* |
|  |  |  |  |  |  |
|  |  |  |  |  |  |
| 406 | Beneficial, gut/external origin | ***Enterococcus*** | **28** |  | ***Enterococcus*** *(****100****, 100)* |
|  |  |  |  |  |  |
|  |  |  |  |  |  |
| 410 | Prevalence | Unkn. *Propionibacteriaceae* | 17 |  |  |
|  |  |  |  |  |  |
|  |  |  |  |  |  |
| 458 | Prevalence |  |  |  | *Oscillospiraceae*:  *Amygdalobacter (****100****, 82)*  Unkn. *Lachnospiraceae* *(17, 99)* |
|  |  |  |  |  |  |
| 466 | Prevalence, **female gland origin** | *Helcococcus* | 20, 41 |  |  |
|  |  |  |  |  |  |
|  |  |  |  |  |  |
| 474 | Prevalence | *Varibaculum* | 2 |  |  |
|  |  |  |  |  |  |
|  |  |  |  |  |  |
| 530 | Prevalence | ***Murdochiella*** | 9, 26, **27**, 28, 43, 49, 53 |  |  |
|  |  |  |  |  |  |
| 534 | Prevalence | ***Fastidiosipila*** | 8, 12, **14**, 15, **18**, 38, 40, 46, 53, 54, **57**, 59 |  |  |
| 538 | Prevalence | ***Unkn. Clostridia* close to *Murdochiella*** |  |  | ***Oscillospiraceae*:**  *Amygdalobacter* *(24, 85)* ***(67)****  *Saccharofermentants (14, 99)*  *Lachnospiraceae*:  *Blautia (18, 96)*  *Coprococcus (20, 93)*  *Roseburia (20, 91)*  ***Peptoniphillaceae*:**  *Parvimonas (20, 88)* |
|  |  |  |  |  |  |
|  |  |  |  |  |  |
| 566 | Prevalence, gut origin, | ***Peptoniphilus*** | **1**, **3**, 34, 58 |  |  |
| 570 | Prevalence | ***Murdochiella*** | 9, **26**, 27, 28, 43, 49, 53 |  | *Oscillospiraceae*:  *Amygdalobacter* *(23, 85)*  *Oscillibacter (18, 88)*  *Saccharofermentants (17, 89)* ***(76)****  ***Lachnospiraceae***:  *Blautia (16, 96)*  *Coprococcus (14, 92)*  ***Peptoniphillaceae*:**  *Parvimonas* *(18, 88)* |
| 566+570 | Prevalence, gut origin | *Unkn. Clostridia* close to ***Lachnospiraceae*** | **10**, **16**, 30, **37** |  |  |
|  |  |  |  |  |  |

***** Cover using Blast-n for the taxa with the highest cover

** High cover of unidentified bacteria from wild bird cloaca samples [2]

REFERENCES

1. Fisher, M.M. and Triplett, E.W. Automated approach for ribosomal intergenic spacer analysis of microbial diversity and its application to freshwater bacterial communities. *Appl Environ Microbiol*, 1999. 65(10): 4630-4636.

2. van Dongen, W.F.D., White, J., Brandl, H.B., Moodley, Y., Merkling, T., Leclaire, S., Blanchard, P., Danchin, É., Hatch, S.A., and Wagner, R.H. Age-related differences in the cloacal microbiota of a wild bird species*.* *BMC Ecology*, 2013. 13(1): 11.

3. Stewart, F.J. and Cavanaugh, C.M. Intragenomic Variation and Evolution of the Internal Transcribed Spacer of the rRNA Operon in Bacteria*.* *Journal of Molecular Evolution*, 2007. 65(1): 44-67.

4. Martín-Platero, A.M., Valdivia, E., Ruiz-Rodríguez, M., Soler, J.J., Martín-Vivaldi, M., Maqueda, M., and Martínez-Bueno, M. Characterization of antimicrobial substances produced by Enterococcus faecalis MRR 10-3, isolated from the uropygial gland of the hoopoe (Upupa epops)*.* *Applied and Environmental Microbiology*, 2006. 72(6): 4245-4249.

5. Martín-Vivaldi, M., Soler, J.J., Martínez-García, Á., Arco, L., Juárez-García-Pelayo, N., Ruiz-Rodríguez, M., and Martínez-Bueno, M. Acquisition of Uropygial Gland Microbiome by Hoopoe Nestlings*.* *Microbial Ecology*, 2017.

6. Martínez-García, A., Martín-Vivaldi, M., Ruiz-Rodríguez, M., Martínez-Bueno, M., Arco, L., Rodríguez-Ruano, S., Peralta-Sánchez, J.M., and Soler, J.J. The microbiome of the uropygial secretion in hoopoes is shaped along the nesting phase*.* *Microbial Ecology*, 2016: 1-10.

7. Soler, J.J., Martínez-García, A., Rodríguez-Ruano, S., Martínez-Bueno, M., Martín-Platero, A.M., Peralta-Sánchez, J.M., and Martín-Vivaldi, M. Nestedness of hoopoes' bacterial communities: symbionts from the uropygial gland to the eggshell*.* *Biological Journal of the Linnean Society.*, 2016. 118: 763-773.

8. Martínez-García, A., Soler, J.J., Rodríguez-Ruano, S., Martínez-Bueno, M., Martín-Platero, A.M., Juárez-García-Pelayo, N., and Martín-Vivaldi, M. Preening as a vehicle for key bacteria in hoopoes *Microbial Ecology*, 2015. 70 (4): 1024-1033.

9. Rodríguez-Ruano, S., Martín-Vivaldi, M., Martín-Platero, A.M., López-López, J.P., Peralta-Sánchez, J.M., Ruiz-Rodríguez, M., Soler, J.J., Valdivia, E., and Martínez-Bueno, M. The hoopoe's uropygial gland hosts a bacterial community influenced by the living conditions of the bird*.* *Plos One*, 2015. 10(10): e0139734.

10. Mariscal-Gijón, P. *Análisis de la población de enterococos en la glándula uropigial de la abubilla europea (Upupa epops)*, in *Zoology*. 2011, Universidad de Granada.

11. Navarro, E., Simonet, P., Normand, P., and Bardin, R. Characterization of natural populations of Nitrobacter spp. using PCR/RFLP analysis of the ribosomal intergenic spacer*.* *Arch Microbiol*, 1992. 157(2): 107-115.

12. Ponsonnet, C. and Nesme, X. Identification of Agrobacterium strains by PCR-RFLP analysis of pTi and chromosomal regions*.* *Archives of Microbiology*, 1994. 161(4): 300-309.

13. Cardinale, M., Brusetti, L., Quatrini, P., Borin, S., Puglia, A.M., Rizzi, A., Zanardini, E., Sorlini, C., Corselli, C., and Daffonchio, D. Comparison of different primer sets for use in automated ribosomal intergenic spacer analysis of complex bacterial communities*.* *Applied and Environmental Microbiology*, 2004. 70(10): 6147-6156.
